# HSP90C interacts with PsbO1 and facilitates its thylakoid distribution from chloroplast stroma in Arabidopsis

**DOI:** 10.1371/journal.pone.0190168

**Published:** 2017-12-27

**Authors:** Tim Jiang, Edward Saehong Oh, Diana Bonea, Rongmin Zhao

**Affiliations:** Departments of Biological Sciences and Cell & Systems Biology, University of Toronto, Toronto, Ontario, Canada; University of California - Davis, UNITED STATES

## Abstract

Arabidopsis plastidic HSP90C is an HSP90 family molecular chaperone that is required for chloroplast development and function. To understand the mechanism of action of HSP90C within the chloroplast, we conducted a yeast two-hybrid screening and revealed it interacts directly with the photosystem II extrinsic protein PsbO1, which performs a canonical function in the thylakoid lumen. To understand the biological significance of HSP90C-PsbO1 interaction, we investigated the role of HSP90C in modulating the stromal and thylakoid distribution of PsbO1GFP fusion protein. Fusion to GFP significantly delays the PsbO1 thylakoid transport and induces a variegation phenotype. Overexpression of HSP90C promotes the thylakoid distribution of PsbO1GFP and alleviates the leaf variegation. By tracking the chloroplast maturation during photomorphogenesis, we observed PsbO1GFP tends to form distinct fluorescent clusters within the stroma with delayed thylakoid membrane biogenesis, while HSP90C overexpression corrects these adverse effects. We also demonstrated that active HSP90C function is specifically required for stable accumulation of mature PsbO1GFP in thylakoid by using specific inhibitor geldanamycin. This study therefore not only identified novel HSP90C interactors, but also reports for the first time that PsbO1 enroute from the cytoplasm to thylakoid lumen is tightly regulated by the HSP90C chaperone complex in plastid stroma; whereas the proper HSP90C homeostasis is also critical for chloroplast maturation and function.

## Introduction

Chloroplast biogenesis is generally characterized by the phenotypic greening of proplastids or etioplasts and *de novo* formation of thylakoid membranes [1]. At the molecular level, this morphological transition requires the cytosolic synthesis of a large set of chloroplast-targeted proteins, as the majority of chloroplast proteins are nuclear-encoded. Upon successful import into the chloroplast, many proteins must undergo folding, assembly, and thylakoid transport for proper function, or immediate degradation. These processes necessitate chloroplast quality control systems that include both molecular chaperones and proteases [2, 3]. Malfunction of protein quality control components have been shown to impair chloroplast function and plant development [4–6]. As an extreme consequence, chloroplast proteins may also undergo bulk degradation through senescence associated vacuoles (SAVs) [7], autophagy [8], or CV (chloroplast vesiculation)-containing vesicles [9], especially under adverse environmental conditions.

HSP90C is an HSP90 family heat shock protein located in the plastid of higher plants and green algae. HSP90C does not show very high similarity to cytosolic homologues [10], particularly at the extreme C-terminal ends [11]. However, it forms a “foldosome” complex consisting of HSP70B, CDJ1 and CGE1 [12, 13], mimicking the cytosolic HSP90 protein complexes required for substrate folding [14]. In the flowering plant Arabidopsis, HSP90C is located in the chloroplast stroma [15] and is required for protein import through the TOC/TIC complex [16]. Seedlings with reduced HSP90C expression caused by transgene-induced gene silencing manifest a variegated phenotype while HSP90C T-DNA insertion homozygous knockouts are embryonic-lethal [11, 16, 17]. Additionally, an early study on point mutation line *cr88* indicated that HSP90C malfunction negatively impacts the expression of light-induced nuclear-localized genes encoding chlorophyll *a/b* binding protein (CAB), small subunit of ribulose bisphophate carboxylase (RBCS) and NR2, resulting in delayed de-etiolation, underdeveloped plastids and yellow cotyledons [18]. These suggest HSP90C has pleiotropic effects in chloroplast maturation and physiology.

HSP90 family proteins generally aid in the late stage of protein folding [19] and its *in vivo* function is revealed by its client proteins and/or cochaperones, which are collectively termed HSP90 binding partners [14]. Both genetic and physical interactors of either the cytosolic or organellar HSP90 isoforms for fungi and human cells have been extensively studied by high throughput analyses [20–25]. However, known interactors of plastidic HSP90C are still limited. Aside from the interactors forming the foldosome [12], HSP90C has been shown to interact with Tic110, Tic40 [16] and VIPP1 (vesicle-inducing proteins in plastid 1) [17]. As a stromal protein involved in protein import through the TIC complex, it is expected that HSP90C may interact with many plastidic proteins. However, because of the developmentally regulated expression [11], it is difficult to study the role of HSP90C in planta.

In order to study the impact of altered HSP90C expression, we previously generated HSP90C overexpression lines and demonstrated that HSP90C overexpression increased the plant sensitivity to salt, osmotic and high calcium stresses [26]. It was also shown that the expression level of HSP90C in young leaves are tightly regulated [11]. To further understand the role of HSP90C in plant growth and development, we performed a yeast two-hybrid screening for Arabidopsis HSP90C interactors in this study. It was identified that PsbO1, a lumen-targeted subunit of photosystem II (PSII) interacts with HSP90C. Additionally, by using PsbO1GFP fusion protein, whose thylakoid transport is delayed compared to native PsbO1, we visualized how this delay negatively impacts chloroplast development *in vivo* and HSP90C homeostasis in chloroplast. We further showed that overexpression of HSP90C alleviates leaf variegation induced by PsbO1GFP overexpression and facilitates thylakoid development. We also analyzed chloroplast maturation during photomorphogeneis and provided evidence that HSP90C level is critical in maintaining chloroplast protein homeostasis and proposed a model of the HSP90C role in guiding PsbO1 targeting from cytoplasm to the thylakoid lumen.

## Results

### Chloroplast stroma-localized HSP90C interacts with lumen-targeted PsbO1

In an effort to screen for Arabidopsis HSP90C interactors and, particularly for potential clients, we performed a yeast two-hybrid analysis using the HSP90C middle and C-terminal domains (HSP90C-MC) as bait and a prey cDNA library constructed from inflorescence tissues [27]. After three rounds of screening, six HSP90C candidate interactors were obtained reproducibly (S1A Fig). Out of these interactors, two proteins encoded by genes At4G23050 (Per-Arnt-Sim domain-containing kinase) and At1G34770 (melanoma-associated antigen protein), were previously reported as AtHSP90C interactors from Arabidopsis Interactome Consortium [28]. The other four interactors are encoded by At5G08170 (agamatine deiminase), At4G21960 (PRXR1, Peroxidase 42), At5G66570 (PsbO1 or oxygen evolving complex 33) and At2G05100 (light harvesting complex B subunit 2, LHCB2). PsbO1 is the major isoform of the two PsbO proteins in Arabidopsis [29]. It is localized in the thylakoid lumen with no known molecular chaperone being previously reported to aid in its chloroplast targeting, thylakoid transport or assembly into photosystem II complex [30]. We therefore chose to conduct in-depth analyses of the HSP90C-PsbO1 interaction.

**Fig 1:**
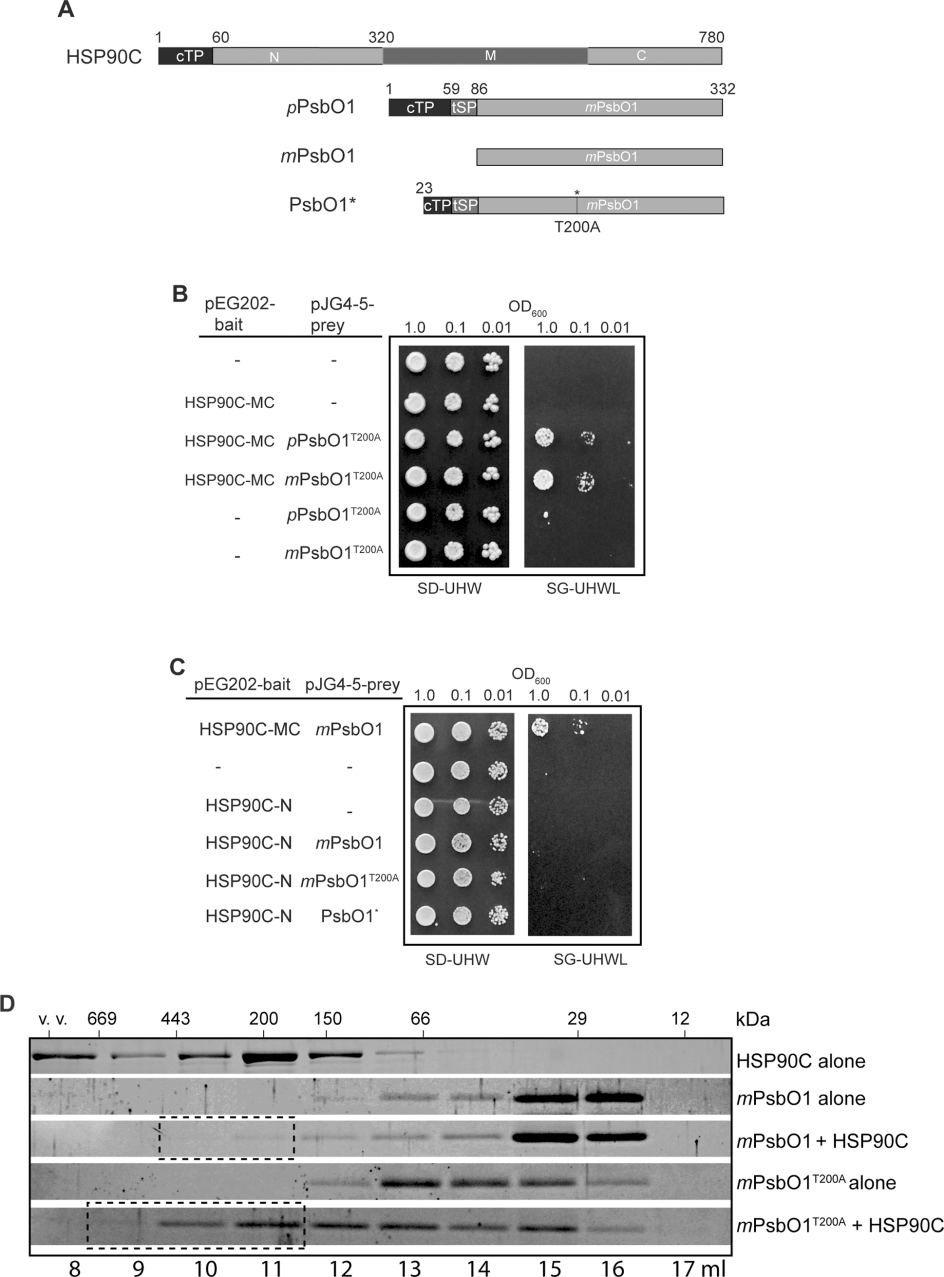
Interaction between HSP90C and PsbO1. (A) Schematic diagram of PsbO1 preprotein (top), mature protein (middle) and the form carrying a T200A point mutation identified in pJG4-5 prey vector (PsbO1*, bottom). (B) Yeast two-hybrid analysis between HSP90C-MC and *p- or m*PsbO1^T200A^ mutant protein. (c). Yeast two-hybrid analysis between native PsbO1 and HSP90C-MC or HSP90C-N. (D)Size exclusion chromatography analysis of *m*PsbO1, *m*PsbO1^T200A^ in presence or absence of HSP90C. Fractions containing PsbO1 bound to HSP90C were highlighted in dashed rectangle boxes.

PsbO1 is nuclear-encoded and the pre-protein contains a chloroplast-targeting peptide and a thylakoid-targeting peptide which are both cleaved after the successful translocation (Fig 1A). Interestingly, the identified *PsbO1* clone in our screen contains only part of the chloroplast targeting sequence and bears a T200A point mutation in the middle of the mature protein (shown as PsbO1* in Fig 1A and S1A Fig). To rule out the possibility that the interaction is mediated only by the chloroplast/thylakoid targeting sequence, we re-constructed the full length and the mature form of PsbO1^T200A^ and confirmed that HSP90C interacts with both the pre-protein and mature forms by yeast two-hybrid (Fig 1B) and co-immunoprecipitation from yeast cell lysates (S1B Fig). We also corrected the T200A point mutation by site-directed mutagenesis and showed that the native PsbO1 mature form interacts with HSP90-MC, while not with the HSP90C N-terminal domain alone (Fig 1C). Further analyses by *in vitro* size exclusion chromatography with purified proteins also indicated that HSP90C shifts the elution profiles of both native and mutant PsbO1 to the high molecular weight range, with an indication that HSP90C interacts more strongly with the mature form of PsbO1^T200A^ (Fig 1D). Collectively, these proteomics and biochemical data demonstrated that the chloroplast stroma-localized HSP90C directly interacts with the chloroplast lumen-localized PsbO1.

### Expression of PsbO1GFP caused delayed cotyledon greening and variegation in vegetative leaves

**Fig 2:**
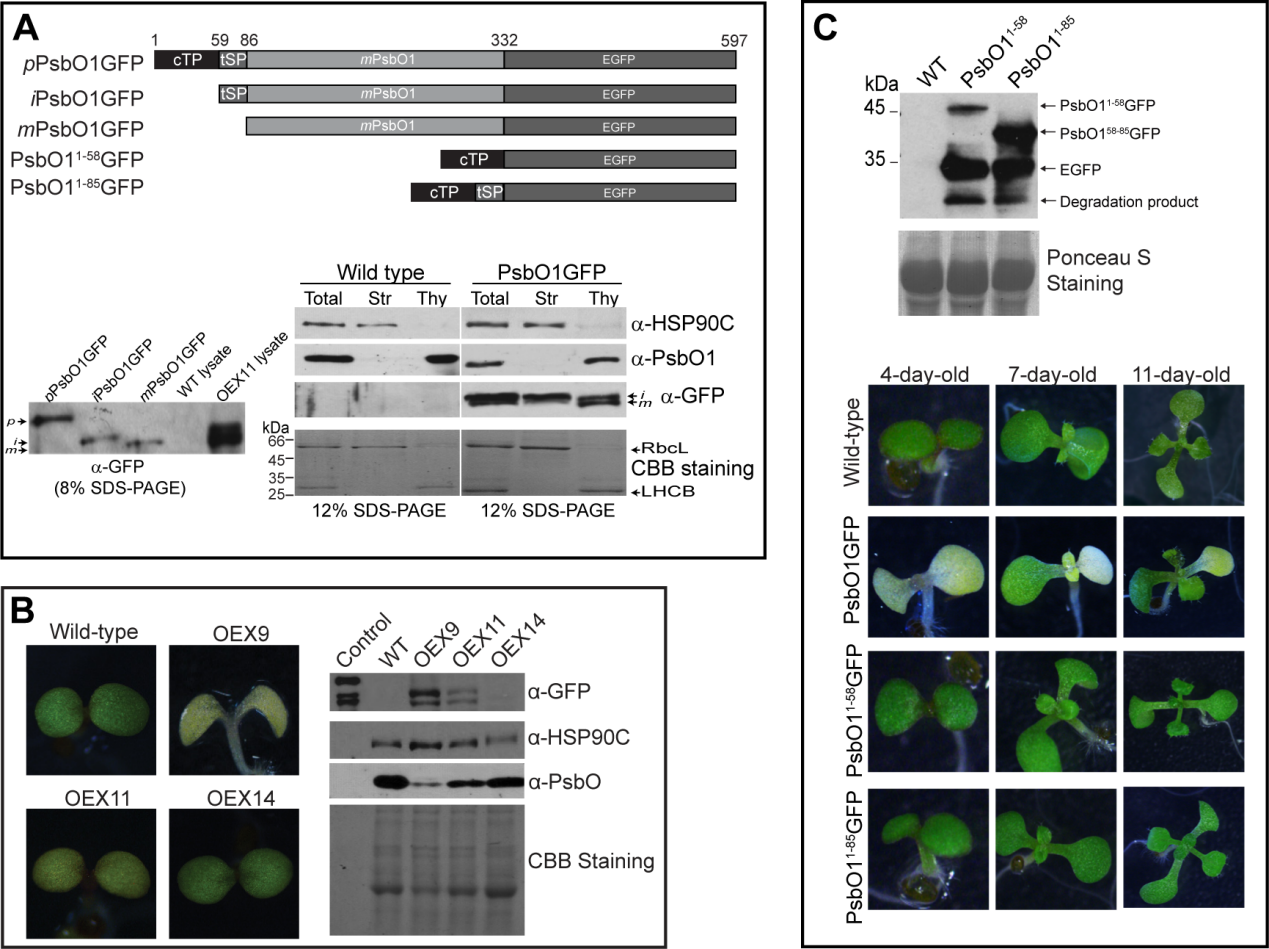
PsbO1GFP transgenic lines show distinct variegation phenotype. (A) Schematic representation of the PsbO1^1-332^GFP, PsbO1^59-332^GFP and PsbO1^86-332^GFP which are also designated as *p*-, *i*- or *m*PsbO1GFP respectively. Control constructs of PsbO1^1-58^GFP and PsbO1^1-85^GFP are also shown. The bottom left immunoblot shows individually expressed *p*PsbO1GFP, *i*PsbO1GFP and *m*PsbO1GFP from *E*. *coli* and, the different processing forms in senescing plant lysate stably transformed with *p*PsbO1GFP (OEX11), which in this study is simply referred as to PsbO1GFP and the Wild type seedling lysate control. The bottom right shows immunoblotting detection of HSP90C, PsbO1 and PsbO1GFP (α-GFP) as well as Coomassie blue staining of stroma (Str) and thylakoid (thy) fractions after fractionation of purified chloroplasts from wild type and PsbO1GFP expressing OEX9 seedlings. Total represents the total chloroplast lysate. (B) T2 seedlings of 3 independent transgenic lines (OEX9, OEX11 and OEX14) expressing PsbO1GFP and wild-type control (Col-0), grown on ½ MS medium for 5 days after germination (5-DAG). Immunoblot analysis was performed with a total of 10 μg total proteins by using anti-GFP, anti-PsbO and anti-HSP90C antibodies. (C) T2 seedlings expressing PsbO1GFP, PsbO1^1-58^GFP and PsbO1^1-85^GFP at 4-, 7- and 12-DAG. The top immunoblot for 2μg of total proteins indicates the expression of PsbO1^1-58^GFP and PsbO1^1-85^GFP in transgenic seedlings by using anti-GFP antibody and Ponceau S staining.

PsbO1 is not well-folded in solution and it is normally rapidly processed in the stroma with only the mature form readily observable in wild-type chloroplasts [29, 31, 32]. PsbO is translocated to the thylakoid lumen in an unfolded state by the SEC complex located in thylakoid membrane [33] To better study the stromal interaction between HSP90C and PsbO1 *in vivo*, we constructed a series of PsbO1-GFP fusion proteins (Fig 2A) that have been shown to delay thylakoid transport in isolated chloroplasts [34, 35]. In order to monitor the processing of PsbO1GFP *in planta*, we also constructed GFP fusion proteins for the full-length pre-protein form (*p*PsbO1GFP), the stromal intermediate form (*i*PsbO1GFP) and the thylakoid lumen-localized mature form (*m*PsbO1GFP) and expressed them in *E*. *coli* for comparison (Fig 2A). The three processing forms of PsbO1GFP expressed in Arabidopsis plants were readily observable (Fig 2A bottom left), indicating delayed chloroplast targeting and thylakoid transport in agreement with previous studies [36]. Fractionation of purified chloroplasts also indicated that *i*PsbO1GFP is mainly trapped in the stromal fraction (Fig 2A bottom right). Interestingly, we observed that seedlings expressing PsbO1GFP showed delayed cotyledon greening with less chlorophyll content (S2 Fig) and the severity of delay depends on the expression level of PsbO1GFP, particularly on the enrichment of stromal *i*PsbO1GFP (Fig 2B, right). Nevertheless, the yellowish cotyledon phenotype gradually disappears as the seedlings develop and grow older (Fig 2C, bottom), suggesting the chloroplast maturation is simply delayed. As control lines, seedlings expressing stroma-targeted PsbO1^1-58^GFP or thylakoid-targeted PsbO1^1-85^GFP (Fig 2A) did not show any observable phenotype compared to wild type seedlings, in spite of high accumulation of GFP or PsbO1^59-85^GFP in stroma (Fig 2C). In addition, we examined a *psbo1* knockout line and did not observe any severe phenotype at the seedling stage under normal growth conditions (S3 Fig) as reported previously [37]. Taken together, these results suggest that overexpression of the full-length PsbO1GFP fusion protein is capable of inducing delayed cotyledon greening.

**Fig 3:**
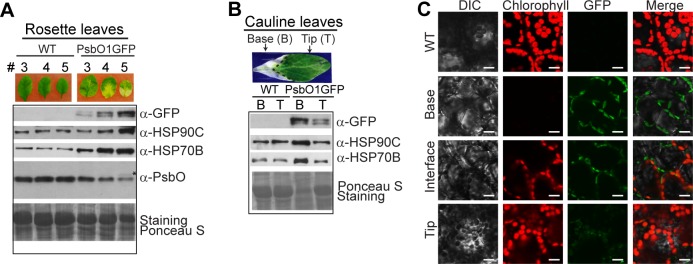
Variegated tissues in PsbO1GFP lines accumulate more stromal *i*PsbO1GFP. (A) and (B) Immunoblot analyses of variegated rosette leaves (A) and the base and tip areas of the first cauline leaf (B) from 35-DAG PsbO1GFP plants. 15 μg of total protein was loaded. *indicate endogenous stromal *i*PsbO1. Anti-GFP, anti-HSP90C and anti-HSP70B antibodies were used and Ponceau S staining was used as a loading control. (C) Fluorescence microscopic analysis of the spongy mesophyll cells from the base, interface and tip regions after peeling. Wild-type was used for comparison. Tip and base regions represent those from 35-day-old cauline leaves as shown in B. Scale bar = 25μm.

At late developmental stages, no significant phenotype was observed for plants expressing PsbO1^1-58^GFP or PsbO1^1-85^GFP. However, plants expressing full length PsbO1GFP show a variegated phenotype in both rosette leaves (Fig 3A) and cauline leaves (Fig 3B). In agreement with the phenotype observed for cotyledons, the severity of the variegation in rosette and cauline leaves is strongly correlated with the expression level of PsbO1GFP, especially the intermediate *i*PsbO1GFP. Surprisingly, the leaf tissues with severe variegation also contain high levels of HSP90C and HSP70B, the two main chaperones of the foldosome [12], suggesting PsbO1GFP in chloroplasts might be mainly bound in the foldosome complex. Nevertheless, the endogenous mature PsbO1 level is significantly reduced in rosette leaves and there seems to be some accumulation of the stroma-localized native intermediate *i*PsbO1 (Fig 3A). We also analyzed mesophyll cells of the cauline leaf using fluorescence microscopy and observed an increase in GFP signal from the green tip to whitened base regions (Fig 3C), further indicating that variegation is caused by accumulation of PsbO1GFP.

To better visualize the sub-plastid localization of GFP signal, we attempted to examine individual chloroplasts in mesophyll cells of the 4-DAG cotyledon. We observed a distinct localization of GFP signal that does not overlap well with chlorophyll autofluorescence in seedlings expressing PsbO1GFP (S4A Fig top). Many plastids form stromule-like extensions, almost like a network containing GFP signal (S4A Fig middle). In some cases, GFP signal was observed in punctate clusters inside these stromules (S4A Fig bottom). We took Z-stack confocal images of the live mesophyll cells and observed that some stromules containing GFP clusters appeared to extend from one plastid and approach or connect to another plastid (S4B Fig). Taken altogether, these results indicate that overexpression of PsbO1GFP might stimulate the formation of stromules, which seem to facilitate inter-plastid transport of the fusion protein.

### HSP90C overexpression alleviates PsbO1GFP overexpression-induced variegation and reduces chloroplast extensions

**Fig 4:**
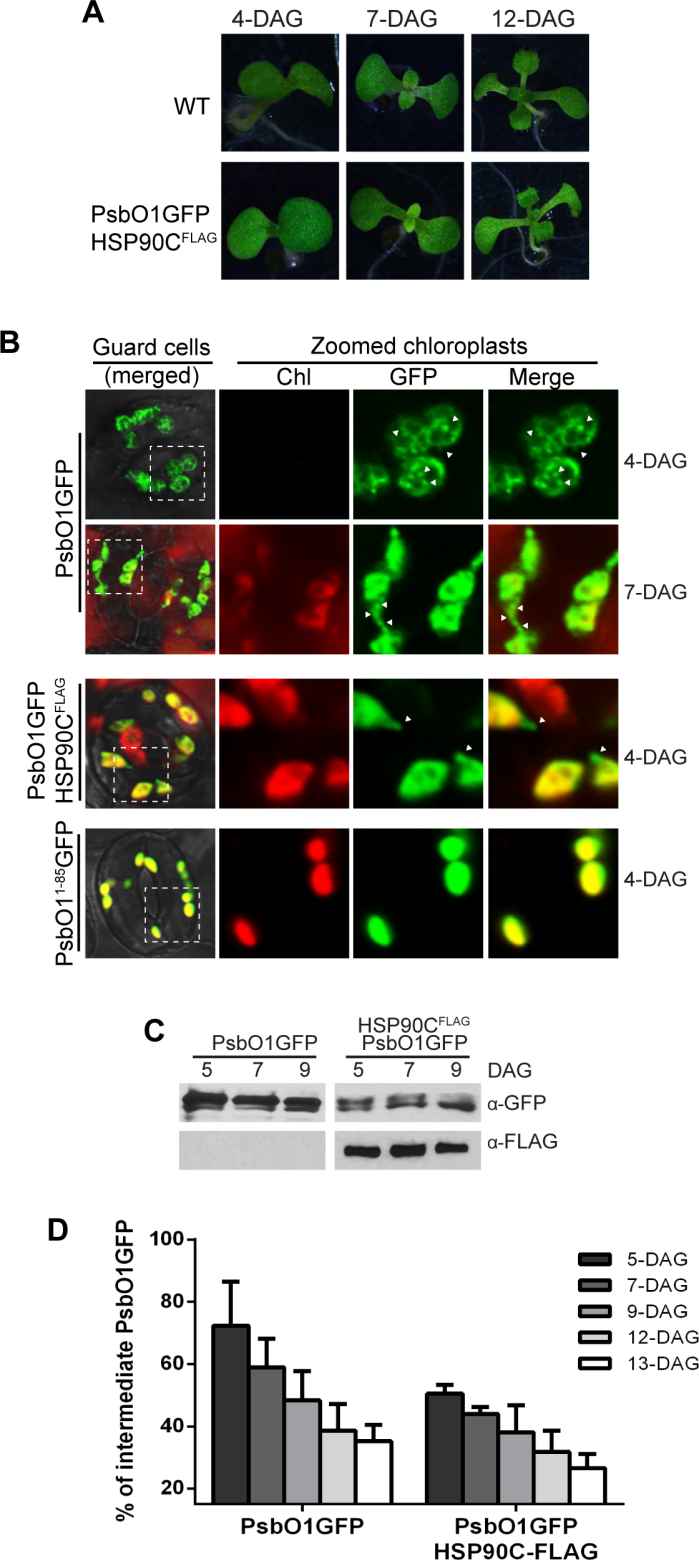
Exogenous HSP90C expression reduced variegation in PsbO1GFP lines. (A) Transgenic lines co-expressing PsbO1GFP and FLAG-tagged HSP90C, designated as HSP90C^FLAG^ (bottom) and wild type seedlings (top) at 4-, 7- and 12-DAG. (B) Confocal fluorescence images of chloroplasts in cotyledon guard cells from PsbO1GFP line, PsbO1^1-85^GFP line and the line co-expressing PsbO1GFP and HSP90C^FLAG^. Samples are from 4- and 7-DAG seedlings. (C) Representative immunoblot analyses of seedlings expressing PsbO1GFP (left), or co-expressing PsbO1GFP and HSP90C^FLAG^ (right) at 5, 7 and 9-DAG. Anti-FLAG was used to indicate the expression of exogenous HSP90C. (D) Quantitative analysis of relative *i*PsbO1GFP signals. Four independent transgenic lines were analyzed for those expressing PsbO1GFP and those co-expressing PsbO1GFP and HSP90C^FLAG^, respectively. The error bars represent standard deviation.

The delayed greening process for PsbO1GFP plants resembles the chloroplast molecular chaperone HSP90C point mutation line *cr88* [15]. However, this phenotype is distinct from that of the HSP90C cosuppression lines for which the variegated phenotype does not disappear over time [11], suggesting the HSP90C function in PsbO1GFP-expressing lines might be only mildly affected. In order to understand the mechanisms by which the greening process is delayed, we transformed the PsbO1GFP construct into Arabidopsis that has been previously transformed with a FLAG-tagged HSP90C [11], designated as HSP90C^FLAG^ lines in this research. Interestingly, analyses of many independent transgenic lines in multiple FLAG-tagged HSP90C transgenic backgrounds did not identify any transgenic plants that have significantly delayed greening process (S1 Table, and Fig 4A). This suggests that overexpression of HSP90C alleviates the PsbO1GFP-induced variegation. We also analyzed guard cells of PsbO1GFP-expressing seedlings. Comparing variegated 4-DAG to less-variegated 7-DAG cotyledons respectively (Fig 4B), we observed that the plastids at 4-DAG have less chlorophyll (Fig 4B) and are swollen, resembling “ballooned” chloroplasts previously observed in *vipp1* mutant lines [38]. Analyses of guard cells of seedlings co-expressing PsbO1GFP and FLAG-tagged HSP90C indicated that they possessed smaller chloroplasts than PsbO1GFP alone and more closely resembled the thylakoid-targeted control line expressing PsbO1^1-85^GFP (Fig 4B and S5 Fig). To further understand how PsbO1GFP-induced variegation develops over time, we analyzed the expression levels of PsbO1GFP in seedlings aged from 5- to 13-DAG. We observed a substantial decrease of the relative amount of *i*PsbO1GFP as seedlings became older and co-expression with HSP90C-FLAG greatly accelerated the drop in *i*PsbO1GFP compared to PsbO1GFP expression alone (Fig 4C and 4D).

**Fig 5:**
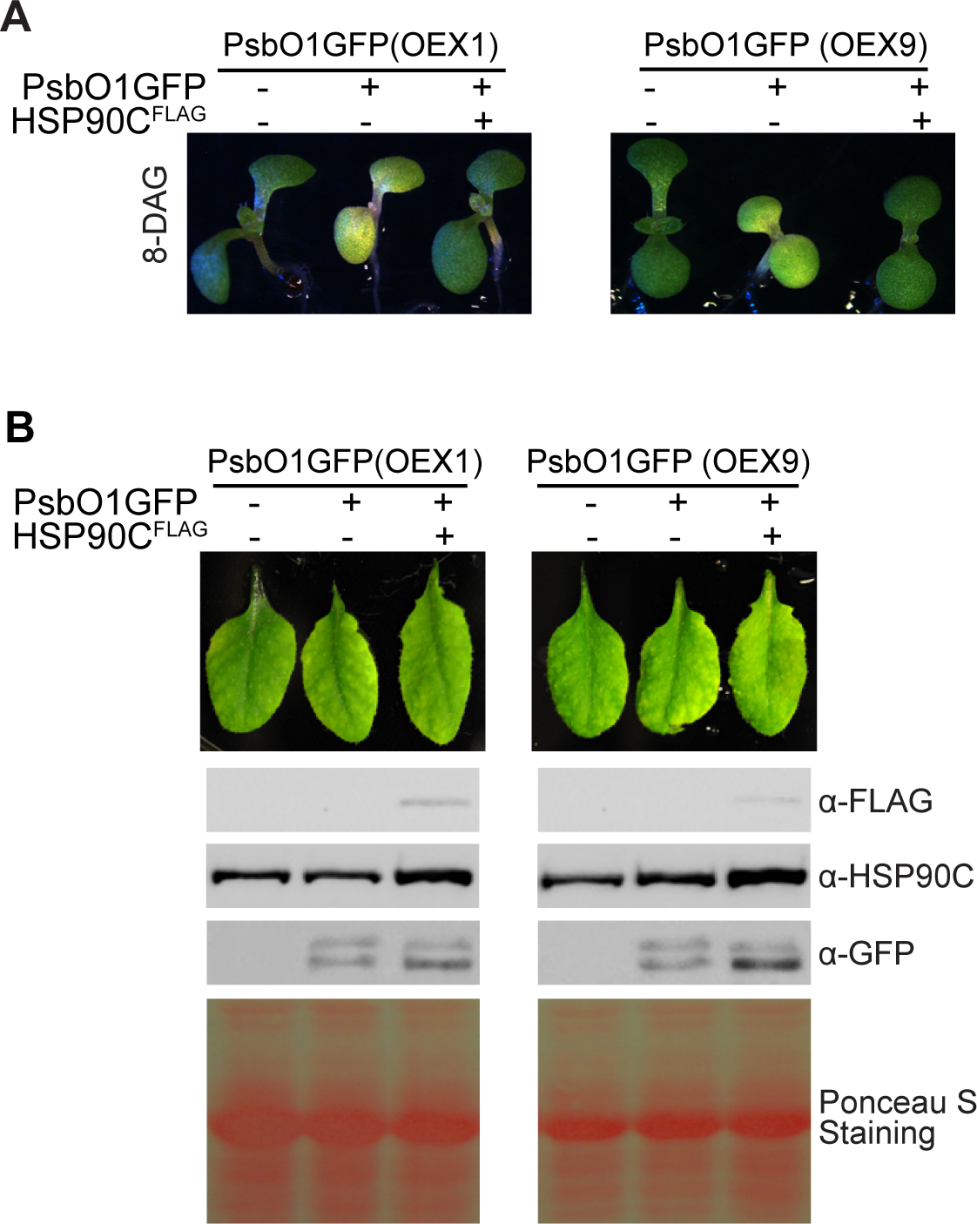
Exogenous HSP90C expression facilitates mature form *m*PsbO1GFP accumulation in leaves. (A) Two independent PsbO1GFP lines OEX1 and OEX9 were crossed with the line expressing FLAG-tagged HSP90C (HSP90C^FLAG^) and propagated to the F3 generation. Siblings expressing PsbO1GFP with or without HSP90C^FLAG^ were identified and grown at 22°C 110μmol/m^2^s and 16h light 8h dark cycle for 8 days. (B) Immunoblot analysis of primary rosette leaves from 41-DAG plants. Protein samples were standardized based on total protein and 10 μg of protein was loaded per lane.

It should be noted that the quantitative data in Fig 4C and 4D are from ten independent PsbO1GFP-expressing lines and four independent PsbO1GFP and HSP90CFLAG co-expression lines, respectively. To confirm that the effect of HSP90C overexpression on PsbO1GFP-induced variegation was not due to the differential expression of PsbO1GFP in analyzed lines, we crossed two independent PsbO1GFP lines with a line that overexpresses HSP90C. The F3 generation plants were obtained and it was shown that the presence of an extra *HSP90C* allele indeed alleviates variegation induced by PsbO1GFP at all developmental stages after germination (Fig 5A and S6 Fig). Interestingly, compared to wild type and HSP90C^FLAG^ co-expressing plants, PsbO1GFP plants developed slightly smaller vegetative leaves. However, their overall development (e.g. transitioning from juvenile to adult and then flowering), was not significantly affected. Particularly, the rosette leaves of PsbO1GFP plants appeared very similar to WT and the HSP90C^FLAG^ co-expression lines after six weeks (Fig 5B). Immunoblot analysis of these rosette leaves indicated that stromal *i*PsbO1GFP levels remain relatively constant with or without HSP90C expression. However, mature *m*PsbO1GFP accumulated to a greater degree in the presence of FLAG-tagged HSP90C (Fig 5B). Taken together, these results suggest that HSP90C helps to reduce the PsbO1GFP-induced variegation and facilitates the thylakoid targeting of mature PsbO1GFP, at least in the mature adult leaves.

### HSP90C expression affects the spatial targeting of PsbO1GFP in chloroplasts

**Fig 6:**
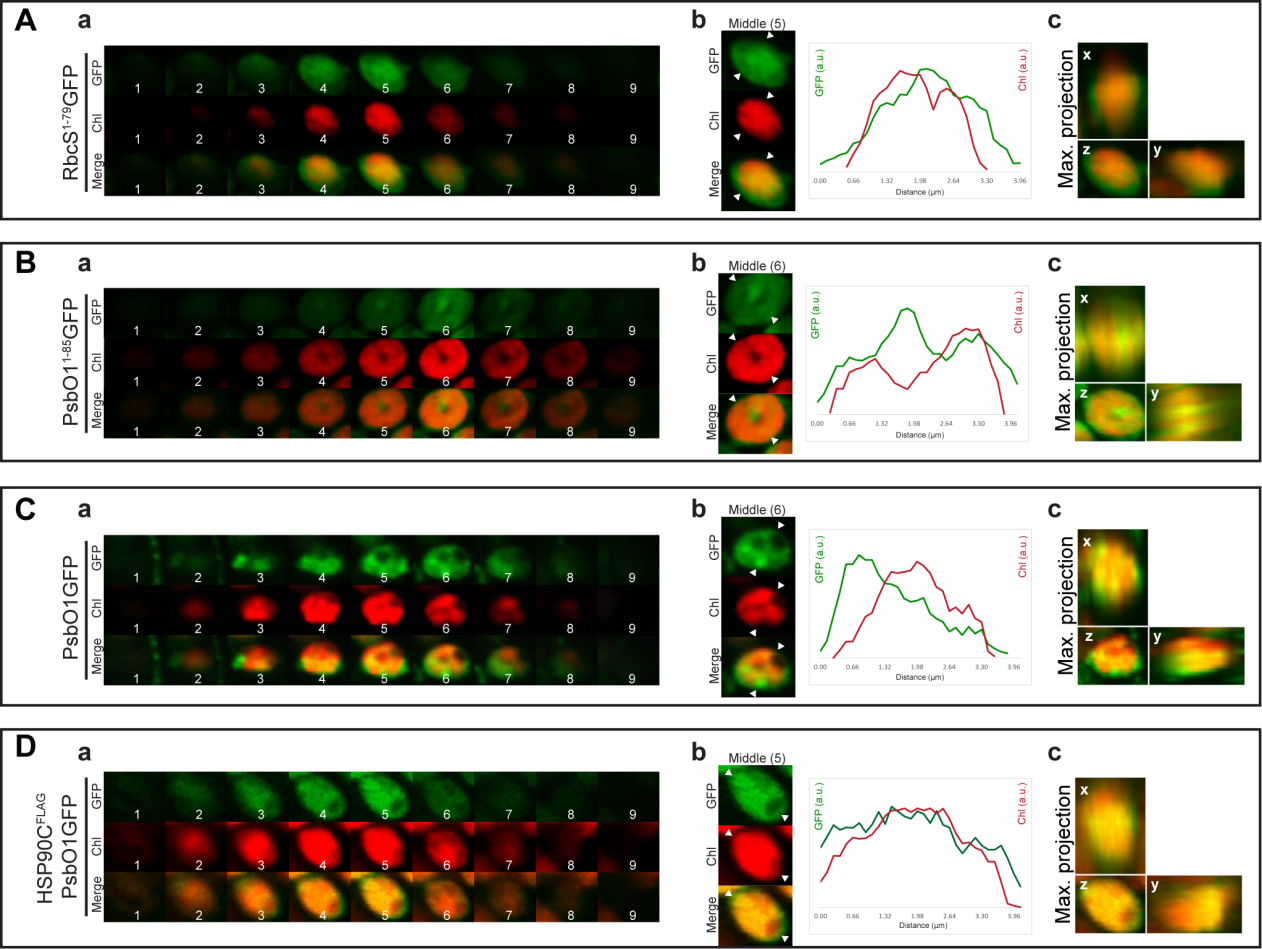
Exogenous HSP90C expression affects PsbO1GFP fluorescence distribution within chloroplasts. Confocal fluorescence images of mesophyll chloroplasts expressing RbcS^1-79^GFP (A), PsbO1^1-85^GFP (B), PsbO1GFP without (C) or with co-expression of HSP90C^FLAG^ (D). Samples are from 4-DAG cotyledons. (a), fluorescence images for GFP, chlorophyll and the merged shown as continuous z-stacks. (b), fluorescence intensity profiles for GFP and chlorophyll analyzed using representative middle slices. (c), the maximum intensity projection of the x-axis, y-axis and z-axis.

HSP90C has been well demonstrated to play a role in protein import through the TOC-TIC complex [16]. However, it is not clear whether HSP90C is directly involved in thylakoid protein transport. To confirm immunoblotting results that show the positive role of HSP90C in affecting PsbO1GFP intermediate and mature form processing (Fig 5B), we analyzed three-dimensional sub-plastid localization of PsbO1GFP when HSP90C is overexpressed using fluorescence microscopy. Chlorophyll autofluorescence, the thylakoid indicator, does not overlap with signal from RbcS^1-79^GFP, a stroma-targeted GFP control (Fig 6A). Conversely, PsbO1^1-85^GFP signal partially overlapped with chlorophyll autofluorescence (Fig 6B) confirming that a portion of GFP protein was successfully targeted to the thylakoid membrane as demonstrated previously by others [34, 35, 39]. GFP signal from full length PsbO1GFP also shows minimal overlap with chlorophyll autofluorescence (Fig 6C), suggesting PsbO1GFP is primarily localized to the stroma. Interestingly, GFP signal from seedlings co-expressing PsbO1GFP and FLAG-tagged HSP90C is mostly overlapped with chlorophyll autofluorescence (Fig 6D), strongly suggesting that overexpression of HSP90C facilitates the attachment or targeting of PsbO1GFP to the thylakoid.

### The effect of HSP90C on etioplast-to-chloroplast development

**Fig 7:**
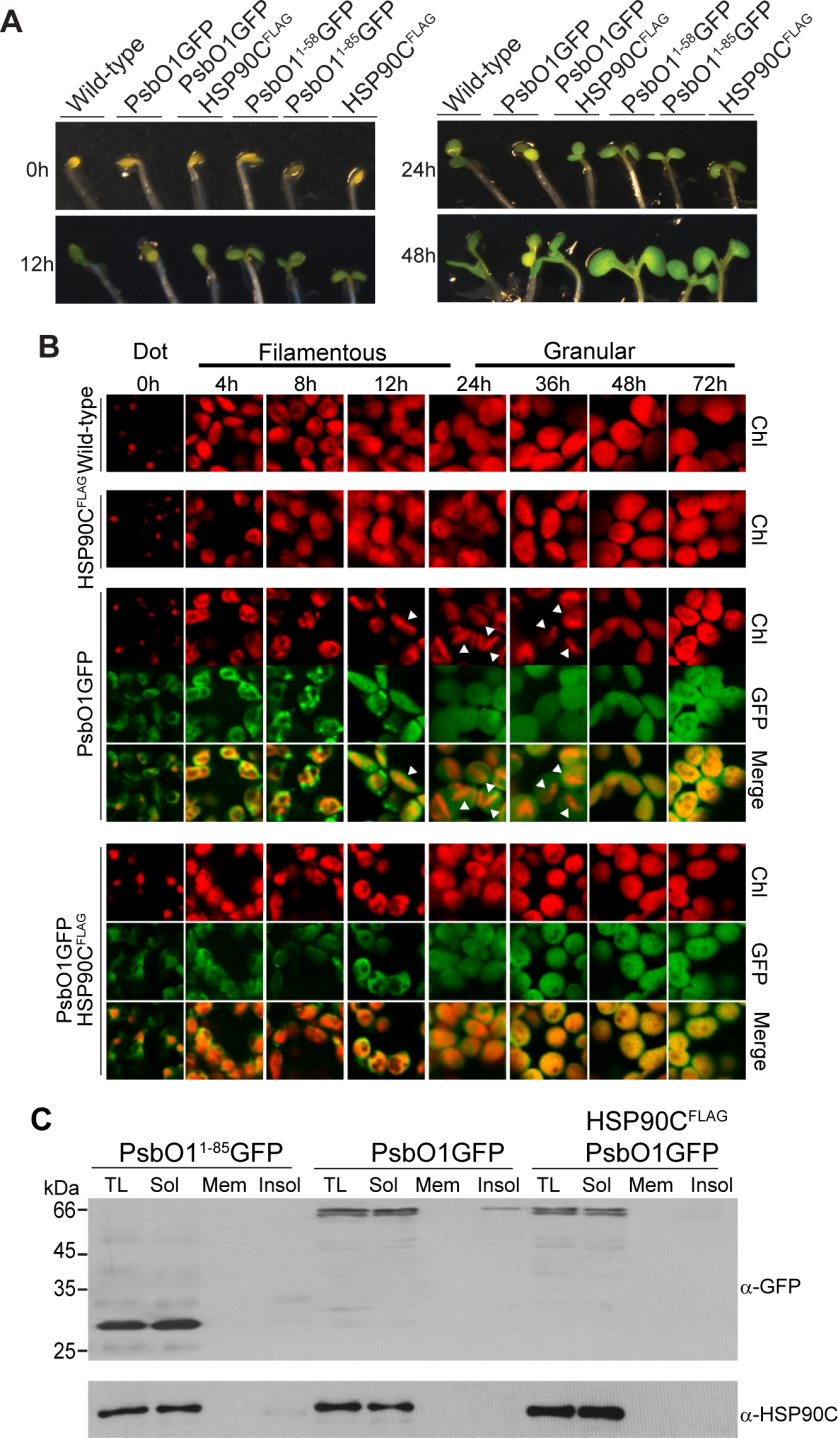
Overexpression of HSP90C affects developmental switch from skotomorphogenesis to photomorphogenesis. (A) Seeds were stratified and grown at 22°C in complete darkness for 3.5 days and then switched to constant light at 110μmol/m^2^s for 48h. Seedling images were taken at 0, 12, 24 and 48h after switching to light. Wild type and lines expressing different reporter proteins were indicated on the top of the seedlings. (B) Confocal fluorescence images were taken for cotyledon chloroplasts in wild type and lines expressing HSP90C^FLAG^, PsbO1GFP without and with co-expression of HSP90C^FLAG^. The white arrows indicate swollen and rod-like chloroplasts. (C) Purification and analysis of insoluble proteins in seedlings expressing PsbO1^1-85^GFP, PsbO1GFP or co-expressing PsbO1GFP and HSP90C^FLAG^. The insoluble fractions were collected after high-speed centrifugation and washed with 2% NP-40. TL, total lysates, Sol, soluble proteins, Mem, membrane bound proteins, Insol, insoluble proteins. The different fractions were loaded equivalently. Anti-HSP90C and anti-GFP were used.

Light is the key factor inducing chloroplast and thylakoid differentiation. When grown in the dark, chloroplast maturation arrests at the etioplast stage as characterized by prolamellar bodies (PLB). Upon exposure to light, the PLB gradually loses its crystallinity and undergoes metamorphosis into primary thylakoids and ultimately thylakoid grana [40, 41]. To understand the chloroplast maturation process and the role of HSP90C on PsbO1 processing during the morphological transition, we germinated different plant lines in the dark and then switched them to constant light, so as to synchronize chloroplasts maturation. It was observed that only PsbO1GFP lines experience dramatically delayed greening, while the other transgenic lines that express HSP90C^FLAG^, PsbO1^1-58^GFP, PsbO1^1-85^GFP, or both PsbO1GFP and HSP90C^FLAG^ green very similarly to the wild type seedlings (Fig 7A).

By tracking the chlorophyll fluorescence, we observed three distinct patterns in wild type and HSP90C^FLAG^ lines over a period of 72 hours after switching to constant light, the “dot,” “filamentous” and “granular” stages (Fig 7B, top) which correspond to prolamellar body, pre-/pro-thylakoid lamellae and grana formation, respectively, as reported in literature [40–42]. The PsbO1GFP line showed a prolonged “filamentous” stage and accumulated less chlorophyll (S7 Fig) within swollen, rod-like structures as opposed to the more uniform dispersal pattern seen in the wild type line. Interestingly, at 8h, distinct punctate of GFP signal appeared within the stromal regions of PsbO1GFP line chloroplasts. However, when HSP90C^FLAG^ was co-expressed, these punctate were scarcely observed (Fig 7B). GFP punctate were also absent from PsbO1^1-58^GFP and PsbO1^1-85^GFP lines, which showed wildtype-like plastidic development overall (Fig 7B).

HSP90 and the other classes of molecular chaperones all have general chaperone activities in preventing client proteins from aggregation under stress conditions. We speculate that some GFP punctate might represent insoluble aggregates form of *i*PsbO1GFP at the pre-/pro-thylakoid stage. We therefore performed a differential centrifugation combined with high concentration of detergent for seedling samples grown 8 hours after switching to light. Indeed, *i*PsbO1GFP was observed in the insoluble fraction of PsbO1GFP line seedlings, with little in membrane fraction which represent membrane bound or vesicle associated PsbO1GFP (Fig 7C). Co-expression of HSP90C caused a striking reduction of insoluble PsbO1GFP protein overall. Nevertheless, it seems that majority of PsbO1GFP is still in soluble form and little or no HSP90C is trapped with insoluble PsbO1GFP (Fig 7C).

### HSP90C interacts with both PsbO1 mature protein and the thylakoid targeting peptide in vivo and is required for PsbO1GFP thylakoid targeting

**Fig 8:**
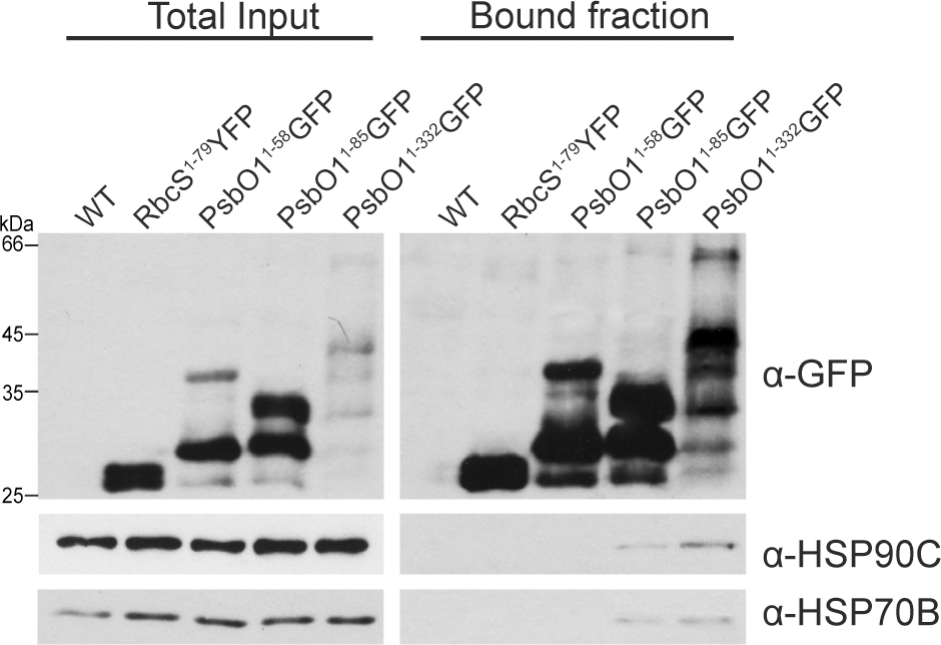
HSP90C interacts and impacts the stroma retention of PsbO1GFP. PsbO1^1-85^GFP and PsbO1GFP were purified with GFP-TRAP beads. Wild-type Col-0 plants (WT), plants expressing RbcS^1-79^YFP and PsbO1^1-58^GFP were used as controls. Both total plant lysate (total input) and bound fractions were analyzed by immunoblotting using anti-GFP, anti-HSP90C and anti-HSP90B antibodies.

We have demonstrated that HSP90C interacts with the mature form *m*PsbO1 using yeast two-hybrid, *in vitro* pulldown and size exclusion chromatography (Fig 1 and S1 Fig). To confirm the interaction in plant cells and to investigate whether HSP90C interacts with the PsbO1 thylakoid targeting peptide alone, we performed co-immunoprecipitation from plant lysates expressing PsbO1GFP and PsbO1^1-85^GFP. Immunoblots showed that HSP90C and HSP70B were co-immunoprecipitated with PsbO1GFP and PsbO1^1-85^GFP, whereas no band was detected with stroma-localized GFP, derived either from RbcS^1-79^YFP or PsbO^1-58^GFP (Fig 8). Additionally, PsbO1GFP was able to co-purify more HSP90C than PsbO1^1-85^GFP, suggesting that HSP90C binds the stromal intermediate form of PsbO1 more tightly than the thylakoid targeting peptide alone. Mass spectrometry analysis of proteins bound to PsbO1GFP confirmed the presence of both HSP90C and HSP70B, together with PsbC/CP43 and PsbS/CP22, which are characterized as a PSII reaction center protein and PSII-associated photoprotection protein, respectively (S2 Table).

**Fig 9:**
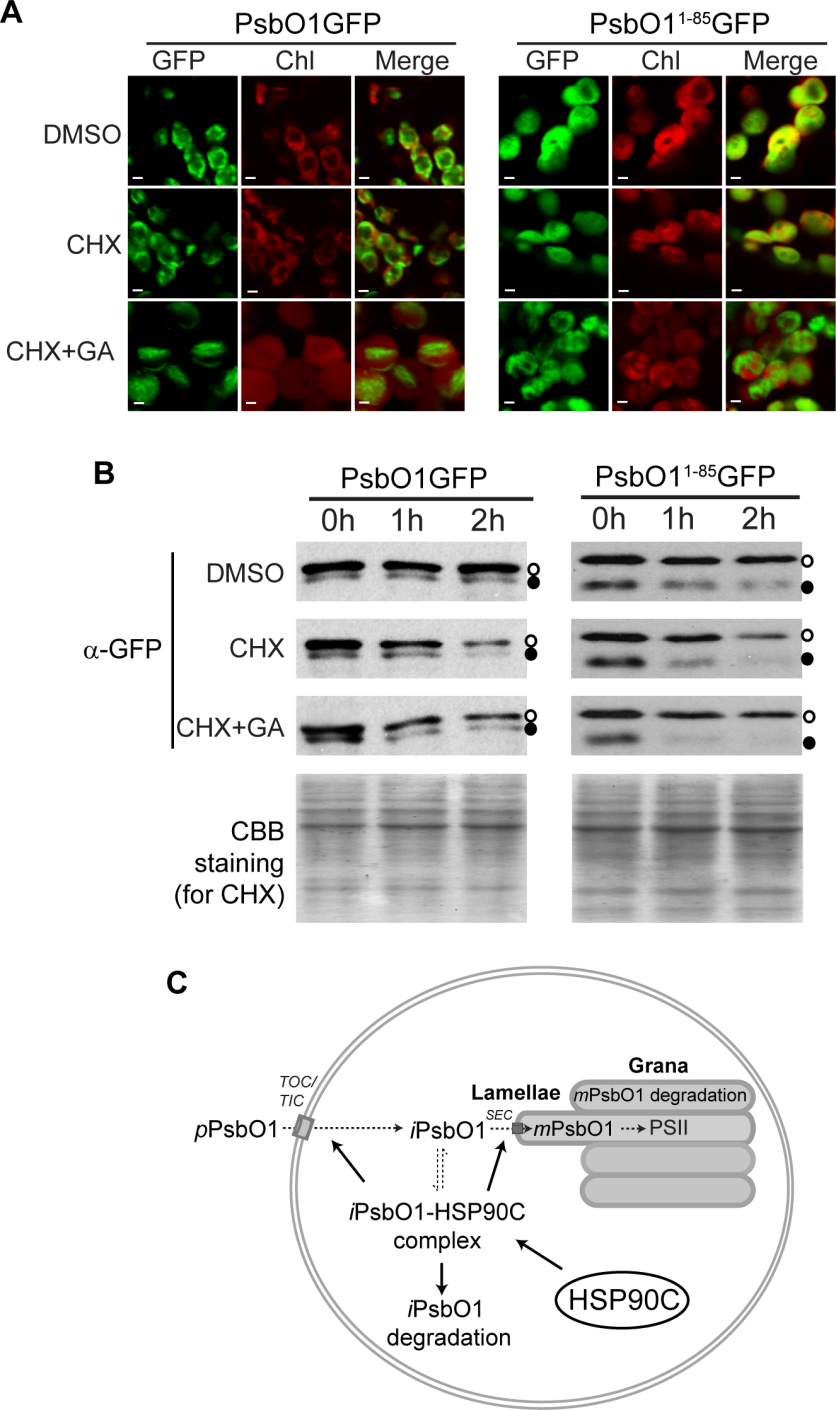
Inhibition of HSP90C activity impairs the thylakoid association of PsbO1GFP. (A) Distribution of GFP fluorescent signal in plastids after inhibition of protein translation using were cycloheximide (CHX) without or with co-application of HSP90 specific inhibitor geldanamycin (GA) for 2 hours. Seedlings expressing PsbO1GFP and PsbO1^1-85^GFP were analyzed. Scale bars represent 2mm. (B) Immunoblotting analyses of intermediate and mature forms of GFP fusion proteins in seedlings treated as shown in **A** for 0, 1 and 2 hours. Anti-GFP antibodies were used and Coomassie brilliant blue (CBB) staining for the CHX treated samples were shown to indicate equivalent loading. Open and closed circles represent the intermediate and mature fusion proteins, respectively. (C) Working model of HSP90C function within chloroplast. PsbO1 is synthesized in cytoplasm and imported into chloroplast stroma through the TOC-TIC complex. PsbO1 is then transported into the thylakoid lumen through the SEC translocon, and integrated into the PSII complex or subjected to degradation immediately. The formation of PsbO1-HSP90C complex in stroma promotes the import, thylakoid transport and likely degradation. Exogenous expression of HSP90C to higher level within stroma promotes the PsbO1-HSP90C complex formation, while the complex formation negatively regulates the availability of HSP90C for other client proteins. The dashed arrows indicate the interconversion of different forms of PsbO1 and the solid arrows indicate the positive role of HSP90C in the processes.

To further understand the role of HSP90C in PsbO1 thylakoid targeting, we treated 5-DAG seedlings with cycloheximide to block protein synthesis in combination with geldanamycin to inhibit HSP90 family protein ATPase activity [43]. It was observed that inhibition of protein translation only caused a reduction of GFP signal as revealed by confocal microscopy (Fig 9A) and rapid degradation of both *i*PsbO1GFP and *m*PsbO1GFP by immunoblotting (Fig 9B). However, the overall distribution of GFP signal was not significantly changed (Fig 9A). Interestingly, co-application of geldanamycin significantly reduced the co-colocalization of GFP signal with chlorophyll autofluorescence (Fig 9A left bottom), suggesting active HSP90C activity is required for thylakoid tethering of PsbO1GFP, or for the active transport of PsbO1GFP into thylakoid lumen where the *m*PsbO1GFP is rapidly degraded (Fig 9B left). Since HSP90C interacts with PsbO1 thylakoid targeting sequence (Fig 8), we also tested geldanamycin on seedlings expressing PsbO1^1-85^GFP (Fig 9A right). Similarly, co-localization of GFP signal with chlorophyll autofluorescence was dramatically reduced as well (Fig 9A right) and the presumably lumen localized GFP protein was degraded rapidly, while the stroma localized intermediate form was relatively stable (Fig 9B, right), thus in agreement with the observation for PsbO1GFP line and suggesting HSP90C activity is required for thylakoid tethering or transport of GFP using the bipartite PsbO1 signal peptide only.

## Discussions

Chloroplasts, being the hallmark of plants, have their own intricate protein homeostasis network that shares very high similarity to its cytosolic counterpart. The sequence and domain structure of plastidic HSP90C is highly conserved [10]. Despite being an essential chaperone for higher plant development and growth, the role and mechanism of action of HSP90C still remains elusive. In this study, we attempted to investigate the Arabidopsis HSP90C interacting partners by yeast two-hybrid screen and obtained a small set of candidate interactors (S1A Fig). This small number may indicate that weak interactions have not been well identified. It should be also noted that a large portion of the proteins within chloroplasts are membrane-associated and the classical yeast two-hybrid screen does not favour the identification of membrane proteins [44]. Screening membrane proteins using the split ubiquitin system [45], or using the recently developed BioID technique [46] could be attempted in the future. Nevertheless, we identified PsbO1 as an HSP90C interactor and confirmed the interaction by a set of *in vivo* and *in vitro* biochemical assays (Figs 1 and 8). In particular, we demonstrated that HSP90C alleviates the variegated phenotype caused by accumulation of PsbO1GFP within stroma (Figs 4 and 5) and facilitates the PsbO1GFP fusion protein tethering/transport to thylakoid (Figs 6 and 9A). This study therefore not only demonstrates a novel role for HSP90C in thylakoid protein transport, but also exemplifies one critical role of HSP90C in maintaining the chloroplast stroma protein homeostasis, which involves protein import from TOC/TIC, transport into thylakoid and degradation in stroma. An integrated model was shown in Fig 9C to represent a scenario mimicking the cytosolic HSP90 which acts as a central hub in the chaperone network [14].

### Active HSP90C protein level is tightly regulated in chloroplasts

Cytosolic molecular chaperones such as HSP90 are generally very abundant under physiological conditions and their expression levels are even higher after heat shock [47]. Arabidopsis plastidic HSP90C, however, is developmentally regulated and not very responsive to heat shock [15]. Reduced HSP90C expression by transgene-induced gene silencing caused a variegation phenotype [11, 17]. This suggests HSP90C level within chloroplasts is well-controlled and there is not much extra HSP90C in the reserve state. In this study, we observed a variegated phenotype for plants that expressed and accumulated high level of intermediate PsbO1GFP (Figs 2 and 3). One scenario could be that PsbO1GFP intermediate forms retained in stroma bind an excess of HSP90C and limit the availability of HSP90C for the other processes, thus mimicking the transgene-induced HSP90C co-suppression plants [11]. However, the variegated phenotype shown in PsbO1GFP plants is different from HSP90 co-suppression lines as the former gradually recovers over time (Figs 2C and S6), while the latter remains variegated. This is reasonable because the steady state level of intermediate PsbO1GFP diminishes over time (Fig 4C). Consequentially, the need for HSP90C is also decreasing and the chaperone can be released from *i*PsbO1GFP to participate in other processes as the plant continues to develop. In agreement with this hypothesis, we noticed high accumulation of HSP70B in chloroplasts as well (Fig 3A) and both HSP90C and HSP70B are associated with PsbO1GFP (Fig 8, S2 Table). This clearly indicates that *i*PsbO1GFP is associated with the HSP90C chaperone complex within the stroma and, non-physiological presence of PsbO1GFP significantly impairs the balance of chaperone network (Fig 9C).

Nevertheless, it should be noted that variegation in green leaves is normally a complicated phenotype and any factor affecting the normal chloroplast biogenesis and maturation may trigger the variegated phenotype [48]. It is technically difficult to identify the cause that primarily triggers the variegation (i.e. by HSP90C cosuppression or by PsbO1GFP expression). Similar variegated phenotypes have been observed for plants with defective function of the other chaperone family members. For example, *Arabidopsis* T-DNA insertion mutants affecting DnaJ-like proteins *wco* [49], *sco1* [50] and *cyo1* [51] also display albino cotyledons as we observed for PsbO1GFP expressing seedlings. Additionally, to understand the molecular mechanism of the variegated phenotype induced by PsbO1GFP expression, re-introducing the different fusion constructs into Arabidopsis with endogenous PsbO knocked out as shown in S3 Fig and analyzing the distribution of the fusion proteins without one of or both the endogenous PsbO proteins may be performed in the future, so as to study the sole effect of exogenous PsbO1 fusion proteins.

### Vital role of HSP90C in thylakoid biogenesis

During embryogenesis, cotyledon cells develop functional green chloroplasts which are later converted to etioplasts at the end of seed maturation [41, 42]. When seeds germinate, etioplasts in cotyledons rapidly differentiate into chloroplasts upon light exposure, while in emerging vegetative leaves chloroplasts are differentiated primarily from proplastids, though mature chloroplasts can also undergo division in certain cells [52]. Thylakoids are characteristic of photosynthetic chloroplasts and therefore chloroplast biogenesis and maturation is marked by the *de novo* thylakoid membrane formation. However, even with extensive studies [53, 54], the mechanism that underlines the thylakoid membrane biogenesis is far from being clearly understood. VIPP1 is a plastid vesicle-inducing protein and required for thylakoid biogenesis [55]. VIPP1 is also required for the thylakoid membrane insertion of PSII complex [56–58]. Interestingly, HSP90C also interacts with VIPP1 [17]. Electron microscopy analysis indicated that the thylakoid membrane is significantly degenerated in both HSP90C co-suppression cells [11] and the HSP90C point mutation, *cr88*, cells [59]. Our yeast two-hybrid analysis also identified that HSP90C interacts with LHCB2, the light harvesting complex protein (S1A Fig). All these studies suggest that HSP90C is a key component in directing thylakoid membrane biogenesis.

Additionally, we examined the photomorphogenesis of seedlings after 3 days of growth in the dark. In agreement with the variegated phenotype observed for seedlings grown under regular light cycles, seedlings expressing PsbO1GFP displayed a significant delay in the greening process that was marked by delayed appearance of chlorophyll (Fig 8). In particular, there are large amounts of green fluorescent clusters in the chloroplasts of cells that express PsbO1GFP only. Co-expression of extra HSP90C in these cells reduces the clusters and enables chloroplast differentiation to proceed as observed in wild type seedlings. It is likely that PsbO1GFP, either in soluble forms or in aggregated clusters, binds and sequesters too much HSP90C, thus causing an imbalance of the chloroplast chaperone homeostasis (Fig 9C).

### Molecular chaperones are required for PsbO1 enroute to the thylakoid lumen

PsbO1 is a thylakoid lumen-localized extrinsic subunit protein of PSII and plays a crucial role in the oxygen-evolving complex by stabilizing the catalytic manganese cluster [60]. The soluble state of PsbO1 has been characterized as a flexible molten globule [61]. It is generally believed that PsbO1 does not require molecular chaperones for folding [62]; rather it folds into native state upon assembly into PSII complex [63–65]. However, we cannot exclude the possibility that PsbO1 does require chaperones during its long journey *en route* to the thylakoid lumen after synthesis in the cytosol. In this study, we provided multiple lines of evidence showing the direct interaction between PsbO1 and the HSP90C foldosome (Figs 1 and 8) and analyzed the role of HSP90C in reducing the PsbO1GFP toxicity (Figs 4, 5 and 7). This clearly indicates that HSP90C may act as a vital regulator for PsbO1 in stroma. Since PsbO1 is not well-folded in solution and is prone to protease attack, it would be plausible to assume the HSP90C foldsome protects PsbO1 from degradation in the stroma and prevents it from aggregating, as our pilot assay revealed an accumulation of insoluble PsbO1GFP proteins at 8 hours after switching to photomorphogenesis (Fig 7). However, we cannot rule out the possibility that association with HSP90C might also facilitate protein degradation, at least for PsbO1GFP, since the steady state level of PsbO1GFP diminishes much more rapidly in the presence of extra HSP90C (Fig 4C). The dual role for cytosolic HSP90 in determining the fate of substrates has been well studied with CHIP E3 ligase [66, 67]. In particular, HSP90C has been previously shown to interact with HSP93, an unfoldase of the chloroplast clpP protease system, in the co-immunoprecipitation experiment [16]. However, we did not identify HSP93 when analyzing the PsbO1GFP complex by tandem mass spectrometry (S2 Table). PsbO1GFP homeostasis and the kinetics of PsbO1GFP degradation within stroma needs to be further investigated.

We initially identified the interaction between HSP90C and a PsbO1 T200A mutant (Fig 1A). It should be noted that T200 is correspondent to T115 with regards to mature PsbO1. This threonine is highly conserved in all PsbO isoforms and we speculate that the point mutation might have arisen during the cDNA library construction and does not exist *in vivo*. The switch from polar amino acid to non-polar alanine seems to slightly increase the strength of interaction with HSP90C (Fig 1D). This is in agreement with the general role of molecular chaperones in binding hydrophobic batches of substrates during folding. It is likely that PsbO1 vigorously interacts with and needs protection from molecular chaperones before it is transported into thylakoid lumen; however, the interaction might be too weak and transient to be visualized by classical biochemical assays. The involvement of stroma chaperonin for thylakoid transport of plastidic type I signal peptidase has been recently reported [68]. By comparing two transgenic lines that bear the same *PsbO1GFP* allele, we showed that extra HSP90C facilitates the accumulation of PsbO1GFP mature form (Fig 5B) and more thylakoid association of GFP signal (Fig 6). Additionally, HSP90C also interacts with the PsbO1 thylakoid targeting peptide (Fig 8). PsbO1 is a native SEC translocon substrate [69]. It would be interesting to investigate in the future whether HSP90C interacts with the SEC complex in thylakoid membrane thus playing a direct role in SEC protein transport.

In conclusion, through yeast two-hybrid assay, we identified photosystem II component PsbO1 as an HSP90C interacting partner and showed evidence that proper homeostasis of HSP90C is critical for chloroplast differentiation and maturation and particularly required for thylakoid transport of PsbO1. We analyzed the growth and development of plants overexpressing PsbO1GFP that has been widely used as reporter protein for chloroplast protein import/transport research, albeit limited study *in planta* particularly at the late development stage. Our study therefore provides a new view of the complicated protein homeostasis network within the unique organelle in plants.

## Materials and methods

### Plant materials and growth conditions

The *Arabidopsis thaliana* ecotype Columbia (Col-0) was used as wild type. To grow seedlings *in vitro*, seeds were surface sterilized and sown on ½ strength Murashige and Skoog (MS) medium containing 1% sucrose and 0.7% agar with or without supplementation by specific antibiotics. After stratification in the dark at 4°C for 3–4 days, seeds were cultured within a plant growth incubator set at 120 μmol.m^-2^.sec^-1^, 16/8 hour light/dark cycle at 22°C. To grow plants to late stage development, seedlings were transferred to soil 10 days after germination for growth within a plant growth chamber set at 110 μmol.m^-2^.sec^-1^, 16/8 hour light/dark cycle at 22°C. RbcS^1-79^GFP, RbcS^1-79^YFP were generated previously [70] and requested from the ABRC.

### Yeast two-hybrid screening

The HSP90C middle and C-terminal domains (aa.320-780), designated as HSP90C-MC was cloned into pEG202 vector for yeast two-hybrid screening as the middle and C-terminal domains bind client proteins [25]. The Arabidopsis cDNA library constructed into prey vector pJG4-5 was a kind gift of Dr. Gazzarrini (University of Toronto, Canada), and the screening of positive interaction using LEU2 and lacZ marker genes was performed in *S*. *cerevisiae* EGY48 cells as previously described [27]. Positive hits were sequenced and retransformed back to yeast cells to test interactions with HSP90C-MC and the N-terminal domain HSP90C-N (aa.61-319).

### Construction of PsbO1GFP fusion genes for expression in *E*. *coli* and plants

The originally identified *PsbO1* gene in pJG4-5 prey vector lacks the coding sequence for the first 22 amino acids and bears a point mutation resulting in Alanine^200^ instead of Threonine^200^, designated as T200A. Site directed mutagenesis was performed to correct the point mutation. An oligo encoding the 22 amino acids was synthesized and used as a primer to construct the full length native PsbO1 gene, which was later cloned into both pJG4-5 and *E*. *coli* expression vector pProEXHTa using EcoRI and XhoI sites. To construct the preprotein, intermediate or mature forms of PsbO1GFP (Fig 1), the coding sequences were amplified by PCR and cloned into the AgeI site of pEGAD vector [71], generating in-frame fusion proteins. A partial digestion for the pre-PsbO1 protein gene was applied when constructing the binary vector. To make chloroplast stroma, or thylakoid targeted GFP constructs using the PsbO1 transit peptide, the coding region for either PsbO1^1-58^ or PsbO1^1-85^ was cloned into the pEGAD AgeI site, creating translational fusion proteins without changing the chloroplast transit peptide or thylakoid targeting peptide cleavage site.

### Arabidopsis transformation and screening of transgenic plants

*A*. *tumefaciens* GV3101 carrying binary plasmid was used to transform Arabidopsis Col-0 by floral dip [72]. Selection of transgenic plants was performed on ½ strength MS medium with 1% sucrose supplemented with 25 μg/mL of glufosinate (Crescent Chemicals), or 25μg/ml kanamycin when needed, and then confirmed by either PCR genotyping or immunoblotting with specific antibodies.

### Protein expression and purification in *E*. *coli*

The constructs for His_6_-tagged mature form HSP90C (aa. 61–780), *m*PsbO1 and *m*PsbO1^T200A^ were made in pProEXhtb plasmid. *p/i/m*PsbO1GFP fusion protein constructs were made by cloning the corresponding coding regions from pEGAD into pET22b vector in NdeI site. Constructs were then introduced into *E*. *coli* BL21 (DE3)-pRIL (Stratagene) and protein expression was induced by 1 mM IPTG. His_6_-tagged proteins were purified using Ni-NTA resin (QIAGEN), and dialyzed overnight. His_6_-tag was cleaved with tobacco etch virus (TEV) protease, removed by Ni-NTA resin and further purified by size exclusion chromatography with a Superdex 200 column (GE Healthcare) in buffer containing 25 mM Tris-HCl, pH 7.5, 150 mM KCl, 10% glycerol, and 0.5 mM DTT.

### Chloroplast isolation and fractionation

The protocol for chloroplast isolation from *Arabidopsis* leaves was adapted from [73]. Briefly, approximately 4 g of 2-week-old *Arabidopsis* seedlings were collected and crushed in 1x homogenization buffer (330 mM sorbitol, 50 mM HEPES-KOH, pH 7.5, 5 mM EDTA, 5 mM EGTA, 1 mM MgCl_2_, 10 mM NaHCO_3_, and 0.5 mM DTT), and filtered through two layers of Miracloth (Calbiochem, CAT# 475855). Intact chloroplasts were collected from the interface after a 30% and 80% percoll gradient centrifugation at 2,000 × g for 10 minutes. Chloroplasts were washed twice with homogenization buffer and saved for total chloroplasts, or further suspended in osmotic lysis HM buffer (20 mM HEPES-KOH pH 7.5, 10 mM NaHCO_3_, 2 mM MgCl_2_, 2.5 mM EDTA, 2.5 mM EGTA, 1 tablet of Roche protease inhibitor in 20 ml) on ice for 15 min. Supernatant (stroma) and pellet (thylakoid) fractions were separated with centrifugation at 2,600 xg for 5min. Pellet fraction was washed three times in HM buffer. Stroma proteins were concentrated with acetone precipitation at 80% final concentration and centrifugation at 10,000xg for 30min. Both stroma and thylakoid proteins were solubilized in 2xSDS buffer with 5 M urea and then subjected to immunoblotting analysis. In experiments that required normalization or estimation of the total chlorophyll *a/b* contents, a formula described in [74] was used after measuring the absorbance at 663 and 646 nm in acetone.

### Immunoprecipitation

To purify GFP or GFP fusion protein complexes from seedlings, seven-day-old seedlings were harvested and ground in liquid N_2_ and then incubated at 4°C for 1h with lysis buffer 25 mM Tris-HCl, pH7.5, 150 mM NaCl, 0.5% NP40, containing protease inhibitor complex (Roche). Co-immunoprecipitation was performed using 25μl GFP-trap resin (ChromoTek) and incubated with cell lysate at 4°C for 1h. The beads were recovered by centrifugation at 1,000xg for 2 min and washed 4 times with 500μl wash buffer 25 mM Tris-HCl, pH7.5, 150 mM NaCl, 0.1% NP40. Bound proteins were released by incubation with 0.1 M glycine, pH2.5 or analyzed using 2xSDS buffer. Eluents were analyzed by SDS-PAGE and immunoblotting or by LC-MS/MS analysis (SPARC BioCentre, Sick Kids, Toronto).

### Fluorescence and confocal microscopy

Fluorescence microscopy was performed using an upright Zeiss LSM 510 confocal laser scanning microscope (Carl Zeiss). The excitation / emission wavelengths were used as following: for GFP, 488 nm/500-530 nm; for CFP, 440 nm/460-490 nm, for YFP, 514 nm/525-552 nm; for chlorophyll, 633 nm/650-720 nm. To avoid overlap between the fluorescence channels, sequential z-stack scanning was used when necessary. Images were processed by ImageJ (National Institutes of Health) or ZEN 2.3 (Carl Zeiss Microscopy GmbH, 2011).

### Fractionation of soluble and insoluble proteins

Possible insoluble protein aggregates were isolated from seedling materials as described [75] with slight modification. Briefly, the seedlings were ground by micro pestle in grinding buffer containing 50 mM MES-NaOH, pH 6.5, 1% glycerol, 0.1% NP40, and then centrifuged at 2,000xg for 5 min followed by 3000xg for 5 min. The supernatant was then centrifuged at 20,000g for 60 min and the supernatant was saved as soluble fraction. The pellet was suspended and washed twice in grinding buffer but with 2% NP40 and supernatant from the first wash was saved as membrane fraction. The final pellet after 20,000 xg for 60 min centrifugation was saved as insoluble fraction.

### Antibodies

Polyclonal rabbit anti-HSP90C antibody was described in [11]. Anti-PsbO1 antibody was generated by Signalway AntiBody (College Park, USA) with purified PsbO1 protein. Other primary antibodies used in this study include anti-FLAG monoclonal antibody (Sigma, F3165) and anti-chloroplast HSP70B (Agrisera). GFP-TRAP resins were purchased from ChromoTek respectively.

## Supporting information

S1 Table Transgenic lines used in this study. (XLSX)

S2 Table PsbO1GFP interactors identified by LC-MS/MS. (XLSX)

S1 Fig Screening and confirmation of HSP90C interactors. (A) Dilution assay of EGY48 strains that carry HSP90.5-MC bait protein and potential interactors. 200 μl of EGY48 cells with optical density at 600 nm (OD600) of 0.1 and 0.01 (indicated on the top) were spotted on synthetic glucose media with triple amino acid dropout for transformation control [(SD-uracil(U), -histidine (H), -tryptophan (W)] and on synthetic galactose media with quadruple drop-out media (SG-UHWL) for interaction test. The plates were incubated at 30°C for 4 days.
(B) Co-purification of HSP90C-MC and *m*PsbO1^T200A^. *In vitro* pulldown of HA-tagged mature PsbO1^T200A^ using anti-HA affinity resin from *EGY48* cell lysate. Immunoprecipitated samples were immunoblotted using anti-LexA antibody or anti-HSP90C to detect the presence of HSP90C-MC protein. Anti-HA antibody was used to test the efficiency of co-immunoprecipitation.
(TIF)

S2 Fig Total chlorophyll contents of independent transgenic seedlings. The concentration of chlorophyll *a* and *b* in extract was calculated by formula (μg/mL) = 20.2 (A645) + 8.02 (A663) after spectrophotometric measurement of the absorbance at 645 and 663 nm.
(TIF)

S3 Fig Seedling phenotype of *psb*O1 T-DNA insertion knockout line. The homozygous psbO1 T-DNA insertion knockout line was confirmed by immunoblotting with anti-PsbO1 antibody. The seedlings were grown for 4, 7, and 11-days.
(TIF)

S4 Fig Transgenic lines expressing PsbO1GFP have more chloroplast extensions that resemble stromules. (A) Top, Expression of PsbO1GFP results in formation of GFP clusters that do not overlap with chlorophyll fluorescence in palisade mesophyll cells. Middle, PsbO1GFP expression was found to induce formation of PsbO1GFP-containing stromule-like extensions. Bottom, smaller plastids above mesophyll chloroplasts were found to contain many small GFP clusters. Scale bar = 2μm.
(B) Stromule-like structures were observed to connect from one plastid to another using z-stack analysis. Each slice is imaged 0.79μm in depth apart. Scale bar = 2μm.
(TIF)

S5 Fig Quantitative analysis of chloroplast size in guard cells. Data are represented as the mean ± STD. n = 100. *p < 0.05; Student’s t-test. Error bars represent standard deviation.
(TIF)

S6 Fig Growth of plants carrying PsbO1GFP with or without HSP90C^FLAG^. Two independent transgenic lines OEX1 and OEX9 were crossed with an HSP90C^FLAG^ overexpression line and propagated to F3 generation. Siblings expressing PsbO1GFP with or without HSP90C^FLAG^ were identified and grown at 22°C 110μmol/m^2^s and 16h light 8h dark cycle for 5 and 12-DAG (**A**), 28 and 35-DAG (**B**) and 41-DAG.
(TIF)

S7 Fig Chlorophyll contents of seedlings after switch from skotomorphogenesis. Seedlings were first grown on MS medium for 3.5 days in the dark and then under constant light. The chlorophyll contents were measured for seedlings grown under light for different times.
(TIF)

S8 Fig Chloroplast maturation under photomorphogenesis. Seedlings were first grown on MS medium for 3.5 days in the dark and then switched to constant light. Confocal fluorescence images were taken for cotyledon chloroplasts in seedlings expressing PsbO1^1-58^GFP (top) and PsbO1^1-85^GFP (bottom).
(TIF)
