# Supplementary figures and images for: HSP90C interacts with PsbO1 and facilitates its thylakoid distribution from chloroplast stroma in Arabidopsis
Source: PLoS One. 2017 Dec 27;12(12):e0190168. doi: 10.1371/journal.pone.0190168 (PMC5745004; doi:10.1371/journal.pone.0190168)

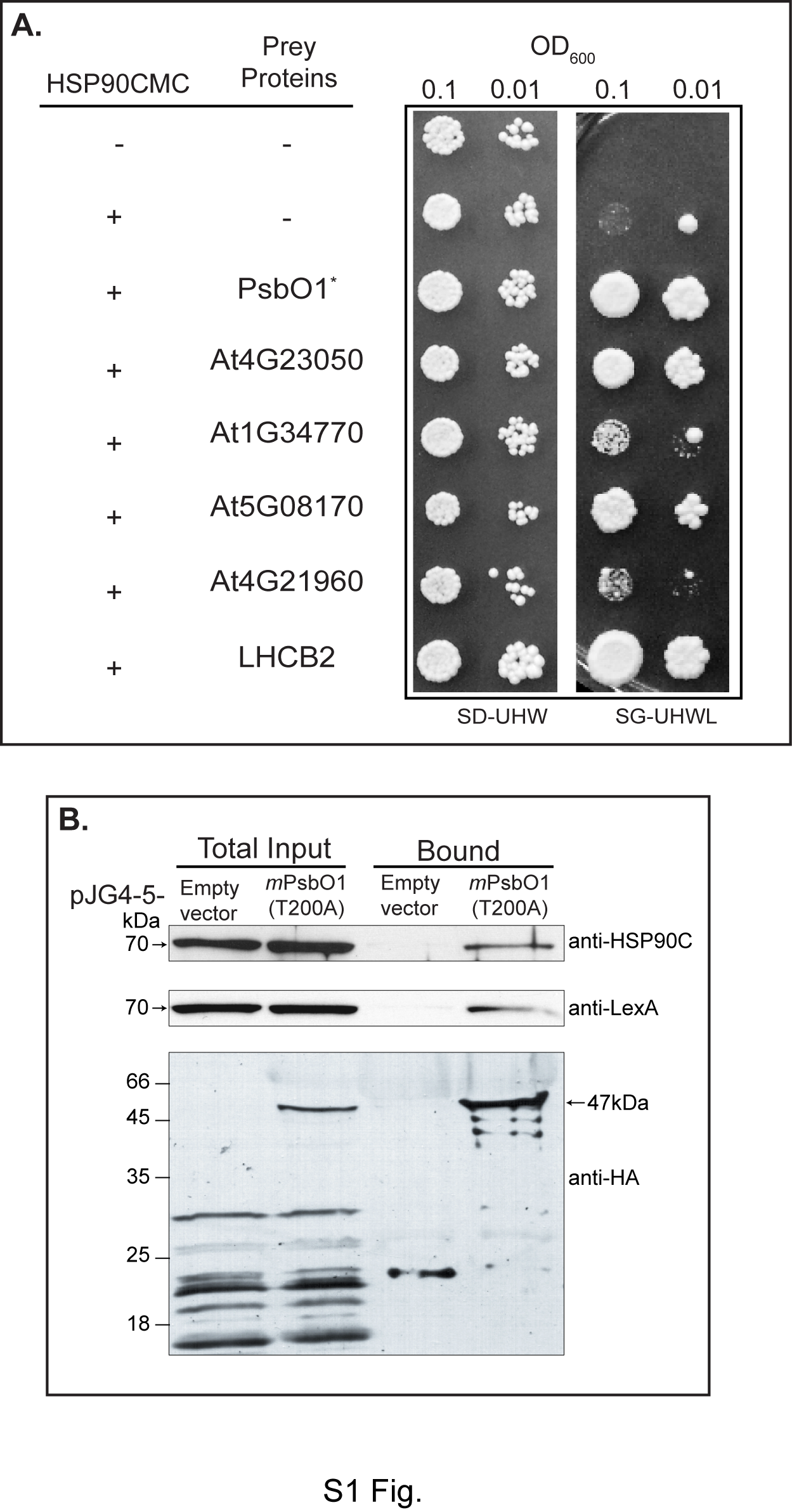

Supplement: S1 Fig — (A) Dilution assay of EGY48 strains that carry HSP90.5-MC bait protein and potential interactors. 200 μl of EGY48 cells with optical density at 600 nm (OD600) of 0.1 and 0.01 (indicated on the top) were spotted on synthetic glucose media with triple amino acid dropout for transformation control [(SD-uracil(U), -histidine (H), -tryptophan (W)] and on synthetic galactose media with quadruple drop-out media (SG-UHWL) for interaction test. The plates were incubated at 30°C for 4 days. (B) Co-purification of HSP90C-MC and mPsbO1T200A. In vitro pulldown of HA-tagged mature PsbO1T200A using anti-HA affinity resin from EGY48 cell lysate. Immunoprecipitated samples were immunoblotted using anti-LexA antibody or anti-HSP90C to detect the presence of HSP90C-MC protein. Anti-HA antibody was used to test the efficiency of co-immunoprecipitation. (TIF) [file pone.0190168.s003.tif]

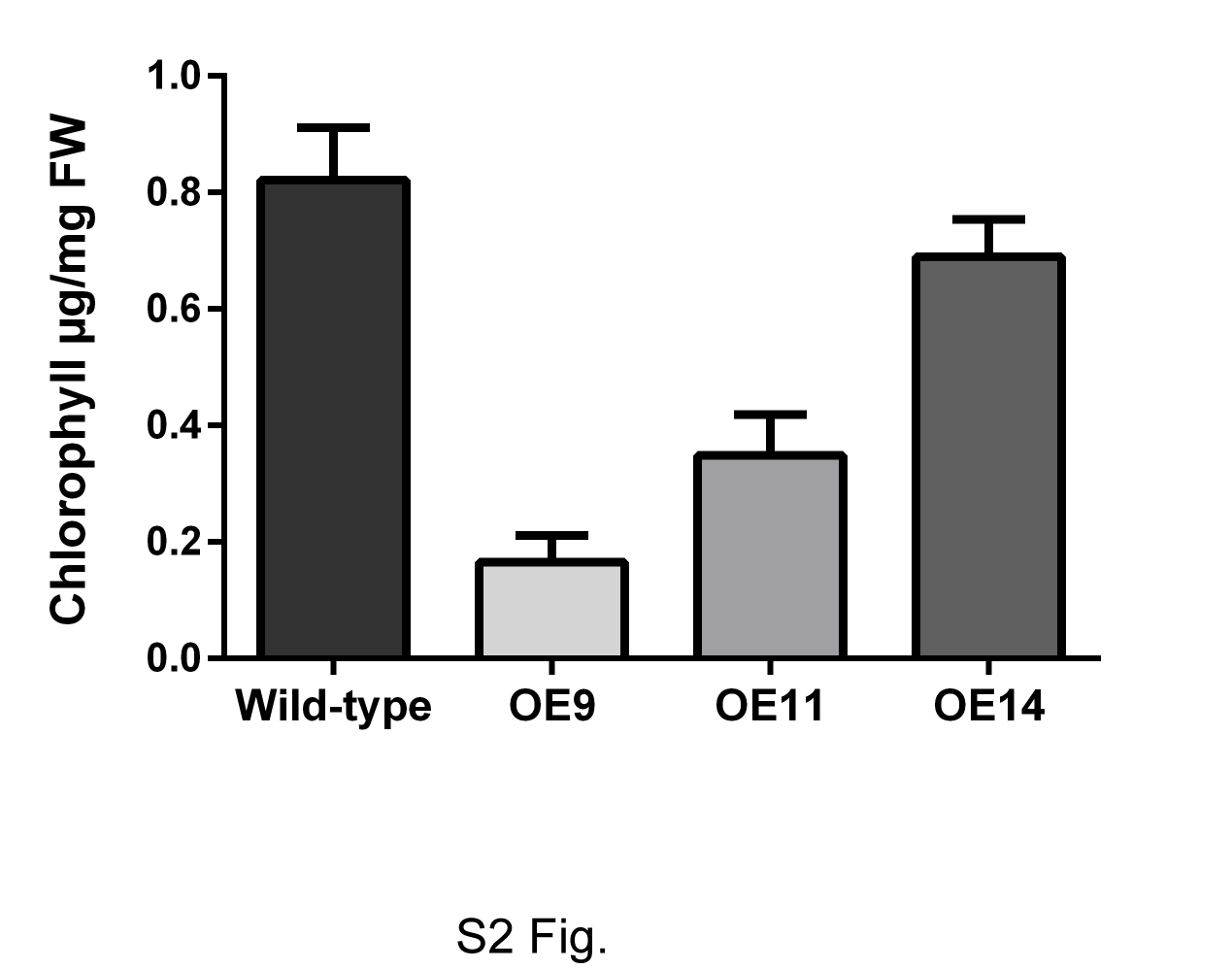

Supplement: S2 Fig — The concentration of chlorophyll a and b in extract was calculated by formula (μg/mL) = 20.2 (A645) + 8.02 (A663) after spectrophotometric measurement of the absorbance at 645 and 663 nm. (TIF) [file pone.0190168.s004.tif]

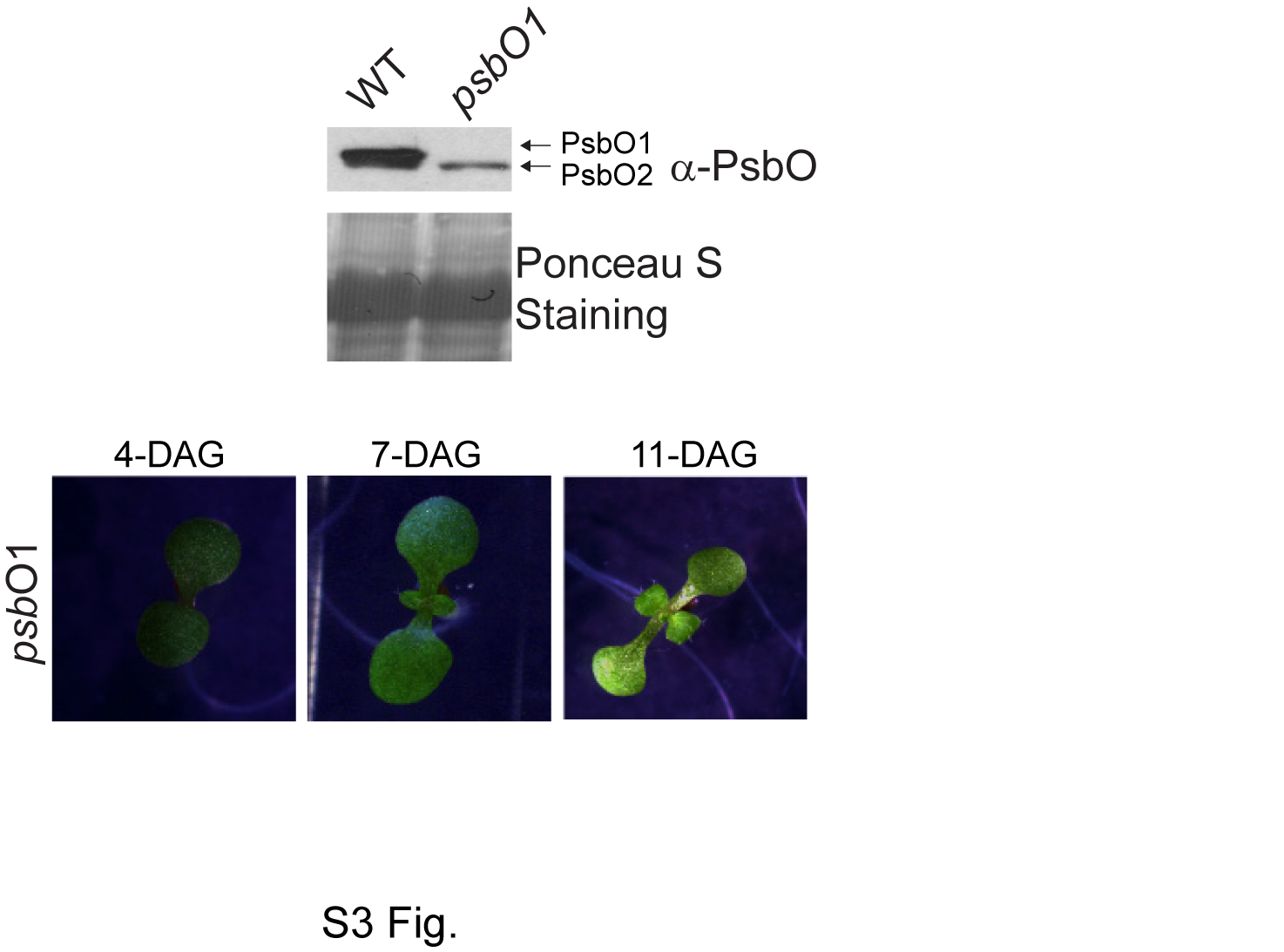

Supplement: S3 Fig — The homozygous psbO1 T-DNA insertion knockout line was confirmed by immunoblotting with anti-PsbO1 antibody. The seedlings were grown for 4, 7, and 11-days. (TIF) [file pone.0190168.s005.tif]

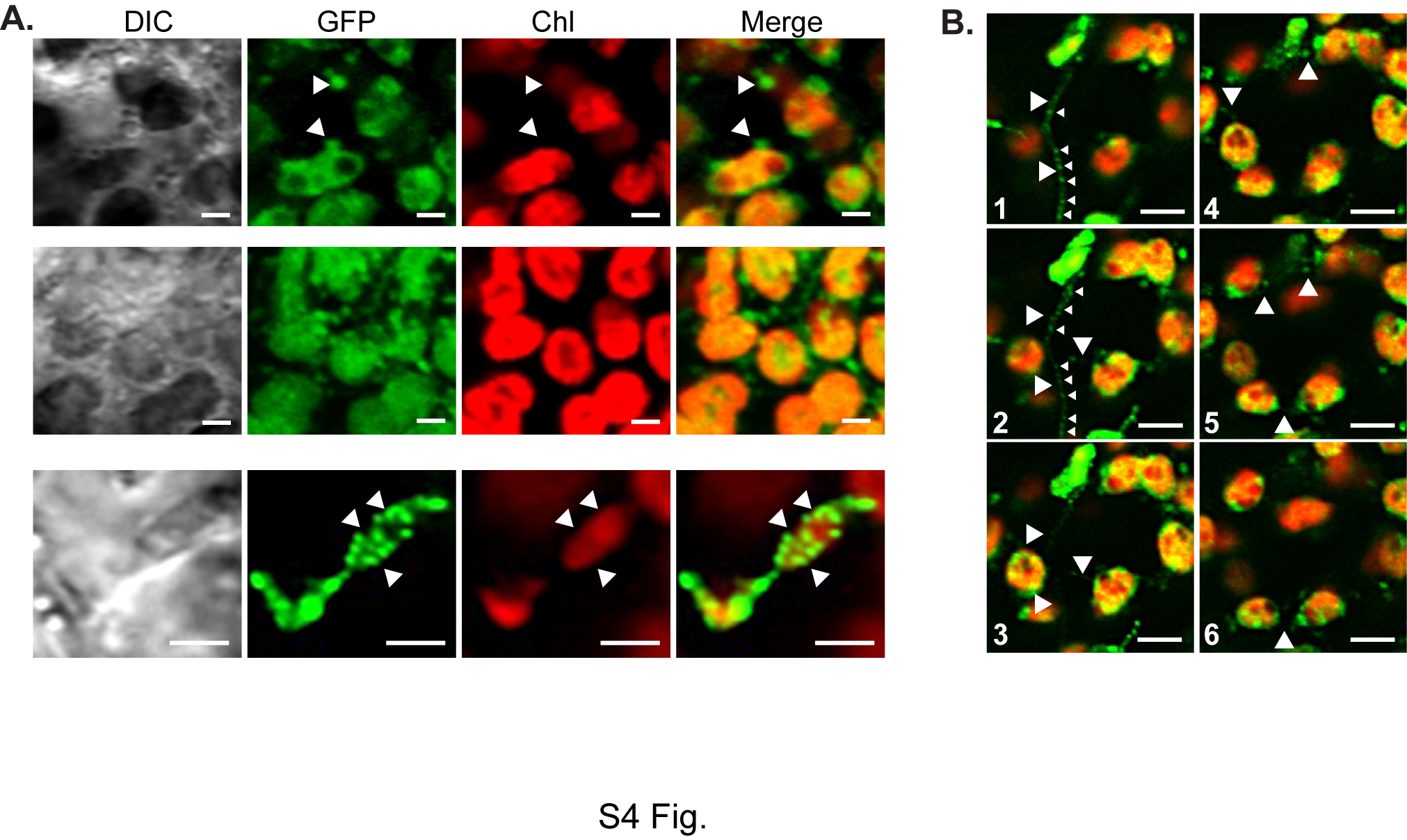

Supplement: S4 Fig — (A) Top, Expression of PsbO1GFP results in formation of GFP clusters that do not overlap with chlorophyll fluorescence in palisade mesophyll cells. Middle, PsbO1GFP expression was found to induce formation of PsbO1GFP-containing stromule-like extensions. Bottom, smaller plastids above mesophyll chloroplasts were found to contain many small GFP clusters. Scale bar = 2μm. (B) Stromule-like structures were observed to connect from one plastid to another using z-stack analysis. Each slice is imaged 0.79μm in depth apart. Scale bar = 2μm. (TIF) [file pone.0190168.s006.tif]

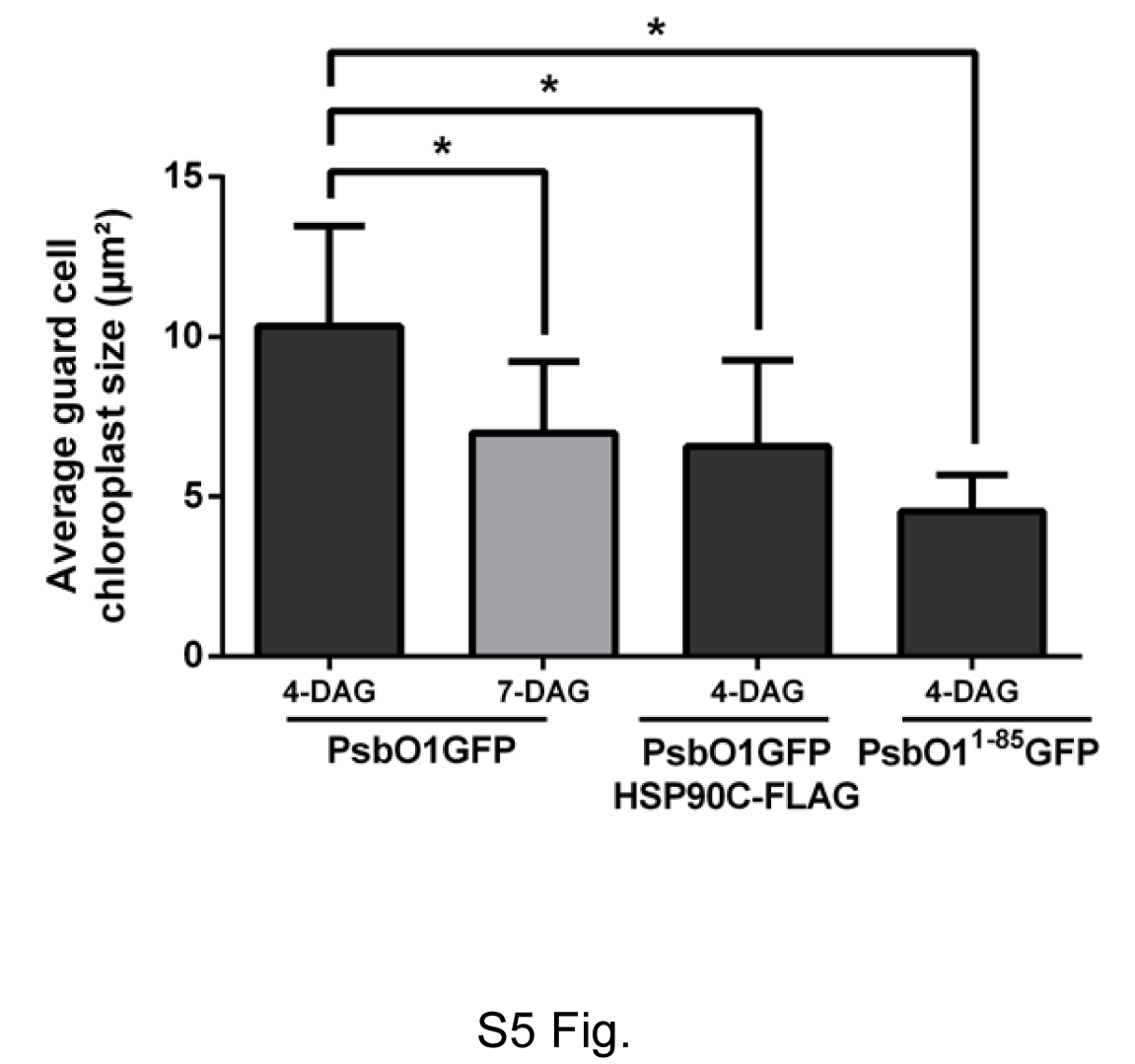

Supplement: S5 Fig — Data are represented as the mean ± STD. n = 100. *p < 0.05; Student’s t-test. Error bars represent standard deviation. (TIF) [file pone.0190168.s007.tif]

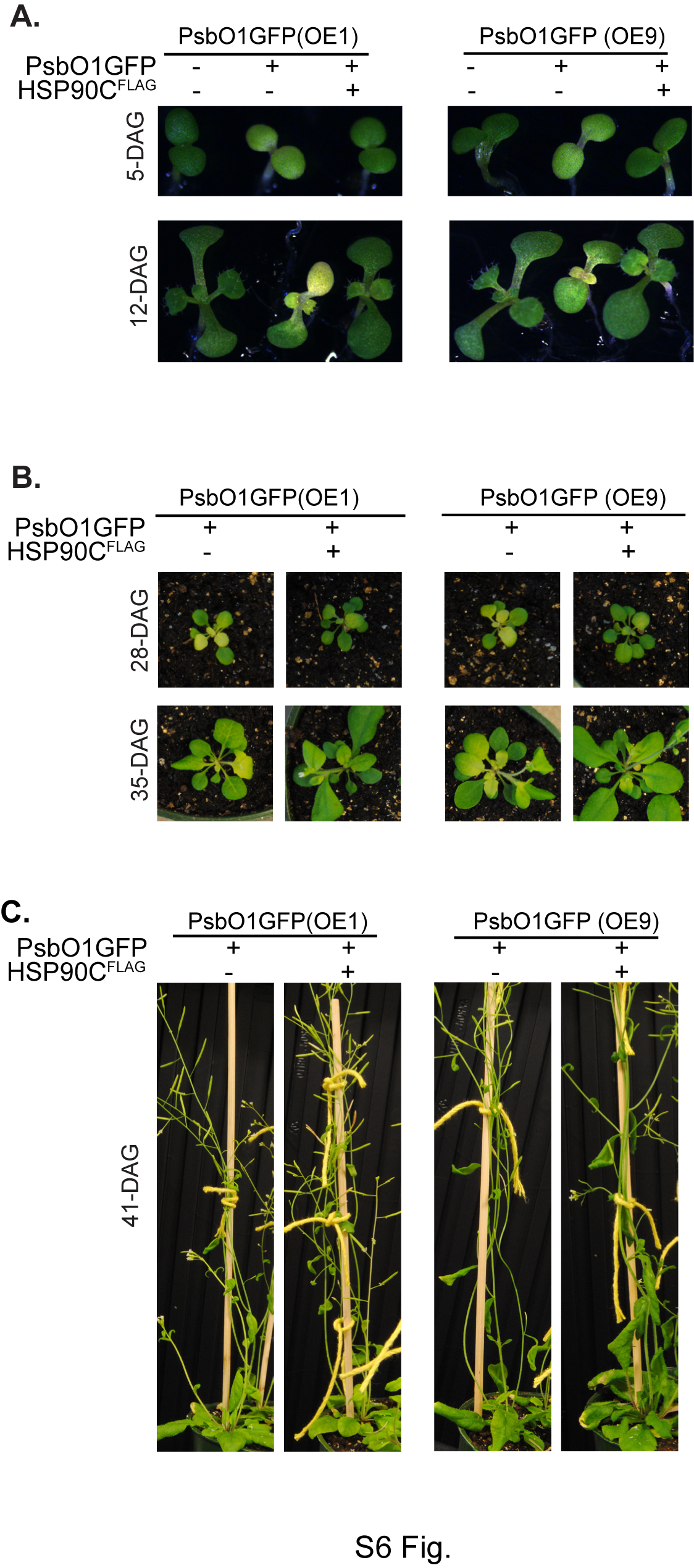

Supplement: S6 Fig — Two independent transgenic lines OEX1 and OEX9 were crossed with an HSP90CFLAG overexpression line and propagated to F3 generation. Siblings expressing PsbO1GFP with or without HSP90CFLAG were identified and grown at 22°C 110μmol/m2s and 16h light 8h dark cycle for 5 and 12-DAG (A), 28 and 35-DAG (B) and 41-DAG. (TIF) [file pone.0190168.s008.tif]

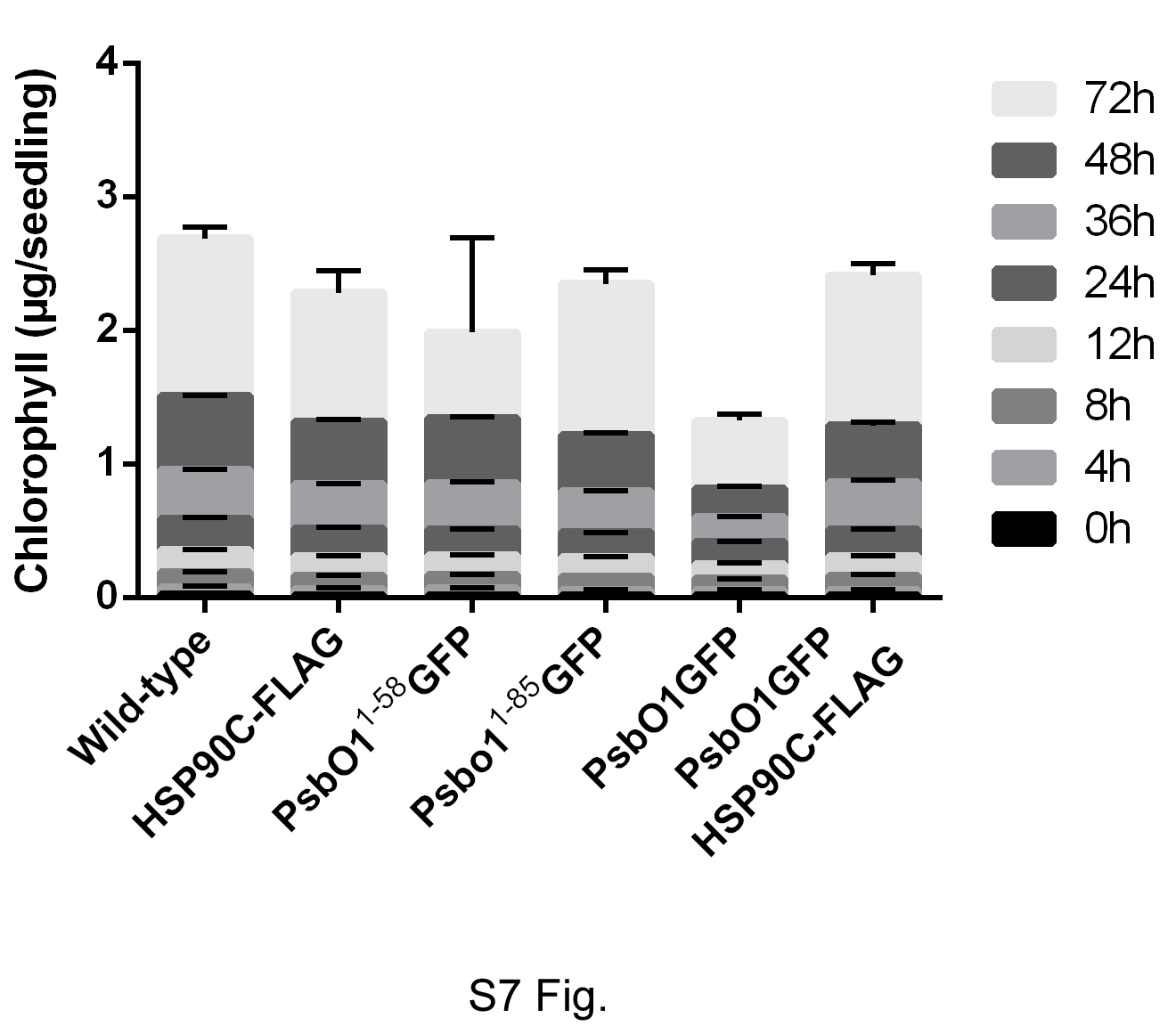

Supplement: S7 Fig — Seedlings were first grown on MS medium for 3.5 days in the dark and then under constant light. The chlorophyll contents were measured for seedlings grown under light for different times. (TIF) [file pone.0190168.s009.tif]

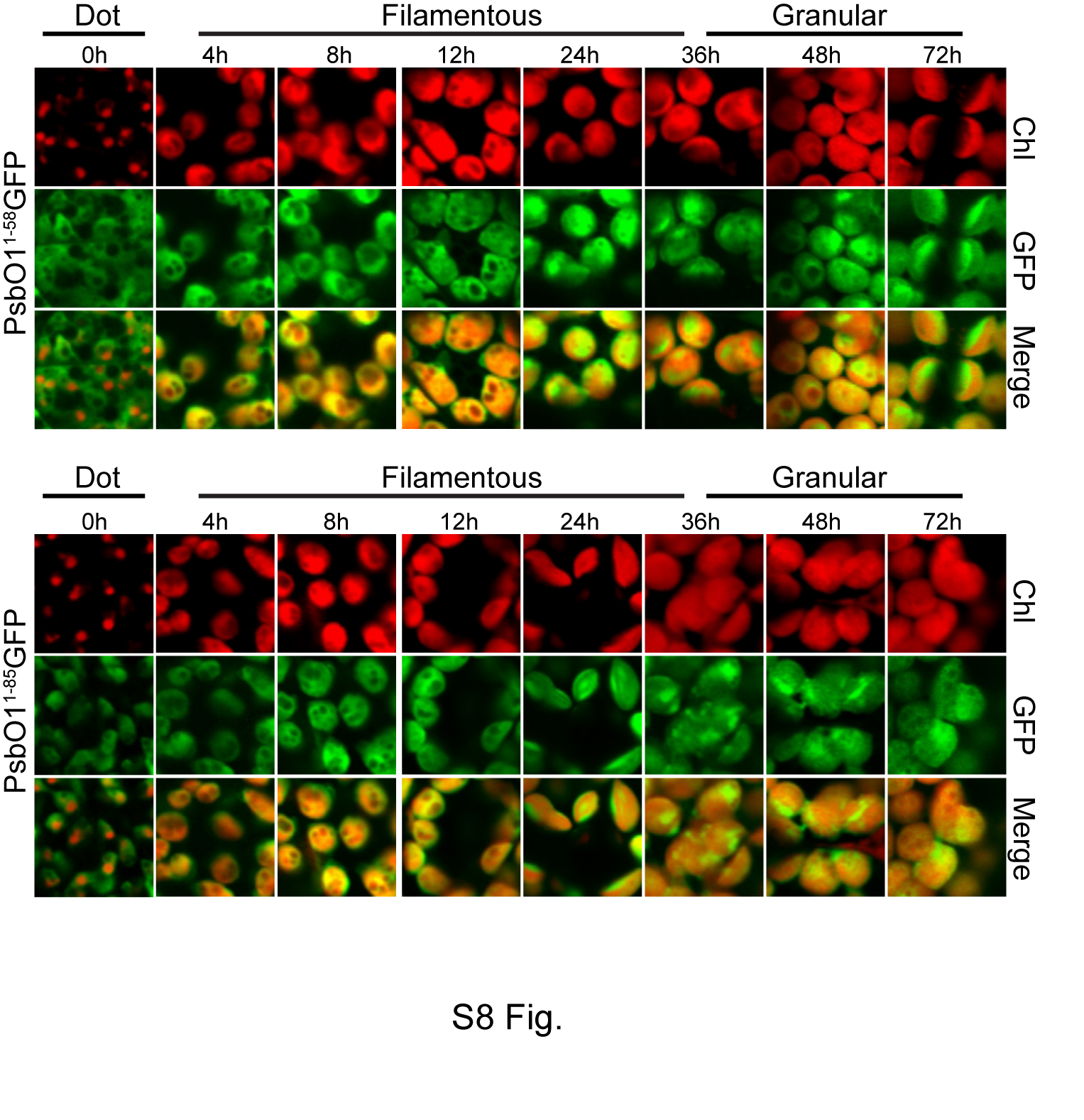

Supplement: S8 Fig — Seedlings were first grown on MS medium for 3.5 days in the dark and then switched to constant light. Confocal fluorescence images were taken for cotyledon chloroplasts in seedlings expressing PsbO11-58GFP (top) and PsbO11-85GFP (bottom). (TIF) [file pone.0190168.s010.tif]
